# Deconvolution of dynamic heterogeneity in protein structure

**DOI:** 10.1063/4.0000261

**Published:** 2024-08-19

**Authors:** Zhong Ren, Xiaojing Yang

**Affiliations:** 1 Department of Chemistry, University of Illinois Chicago, Chicago, Illinois 60607, USA; 2 Renz Research, Inc., Westmont, Illinois 60559, USA; 3 Department of Ophthalmology and Visual Sciences, University of Illinois Chicago, Chicago, Illinois 60607, USA

## Abstract

Heterogeneity is intrinsic to the dynamic process of a chemical reaction. As reactants are converted to products via intermediates, the nature and extent of heterogeneity vary temporally throughout the duration of the reaction and spatially across the molecular ensemble. The goal of many biophysical techniques, including crystallography and spectroscopy, is to establish a reaction trajectory that follows an experimentally provoked dynamic process. It is essential to properly analyze and resolve heterogeneity inevitably embedded in experimental datasets. We have developed a deconvolution technique based on singular value decomposition (SVD), which we have rigorously practiced in diverse research projects. In this review, we recapitulate the motivation and challenges in addressing the heterogeneity problem and lay out the mathematical foundation of our methodology that enables isolation of chemically sensible structural signals. We also present a few case studies to demonstrate the concept and outcome of the SVD-based deconvolution. Finally, we highlight a few recent studies with mechanistic insights made possible by heterogeneity deconvolution.

## INTRODUCTION

When Keith first introduced time-resolved crystallography to us in the early 1990s, he attracted our attention to a couple of challenges in data analysis, in addition to several scientific problems in biochemistry that would significantly benefit from such technical advances in data processing. One challenge was the crystallographic data reduction from the photographs of polychromatic Laue diffraction (Ren and Moffat, 1994), then commonly recorded on negative films and phosphor imaging plates. The other was the resolution of structural heterogeneity intrinsic to the dynamic process of a biochemical reaction or a protein conformational change—the kind of process that time-resolved crystallography aims to study. During those years when we were students, postdocs, and research associates in Moffat lab, we had gained firsthand experience in dealing with mixed signals in our data. Later in our independent research careers, solving these computational problems became one of our goals. As we demonstrate in this review, proper treatment and resolution of structural heterogeneity directly impact structural findings, therefore scientific conclusions.

Prior to the first wave of time-resolved crystallographic studies largely based on the Laue diffraction technique (Bourgeois and Weik, 2009; Ren *et al.*, 1999; and Schmidt, 2008), Moffat laid out the premise from the outset that “the evolution of the system is completely stochastic; there is no temporal coherence between the molecules, and molecules of a given conformation are distributed spatially at random in the lattice.” He further pointed out that the independence of each protein molecule in a crystal lattice arises from “the weakness of the intermolecular forces” (Moffat, 1989). After practicing for a decade, Moffat reflected on the challenge and opportunity in time-resolved crystallography (Moffat, 2001): “The changes in structure from one molecule to another are uncorrelated in space and time …. One molecule is in-state [*i*] does not affect the probability that its neighbors in the crystal lattice are also in-state [*i*] or in any other state [*j*].” Time-resolved crystallography, as Moffat proposed, essentially takes advantage of such coexistence of multiple states. “Time-resolved experimental approaches do not attempt chemical or physical trapping of intermediates, accept that chemical and structural heterogeneity is inherent in the sample at all times as the reaction proceeds, and attempt to resolve this time-dependent heterogeneity into the structures of homogeneous intermediates during the subsequent data analysis process.” However, he also predicted that “… resolution of structurally heterogeneous data into an overall mechanism and time-independent structures of intermediates is a challenging conceptual, structural and computational problem.” In a nutshell, Moffat proposed an elegant method without the need for any deliberate experimental trapping to simultaneously determine metastable reaction intermediates that could rather be called “time-unresolved crystallography.” Despite Moffat's articulation on the meaning of “time-resolved crystallography,” this phrase is often interpreted literally in many high-profile publications with merely a layman understanding that two well-resolved intermediate states can be unconditionally observed by electron density maps measured at two different reaction times *t*_1_ and *t*_2_ regardless of the limited resolving power of time (see below).

Observations in time-resolved crystallography are a collection of structure factor sets at various time points *t* along the pathway of a biochemical reaction. Each structure factor is denoted as ***F***(*t*, ***H***), where ***H*** = (*h*, *k*, *l*) is a triplet of Miller indices, or identification of each diffracted x-ray, within the limit of spatial resolution. While the amplitude of the structure factor is measured in a time-resolved experiment, the phase is usually inherited from a known reference structure and could be further improved using the technique of isomorphous noise suppression (INS) similar to solvent flattening (Ren *et al.*, 2001). Moffat asserted that these time-resolved observations can be represented as linear combinations of structural factors of multiple states:

$$F\left(t,H\right)=\sum_{i}f_{i}(t)F_{i}(H),$$ (1)

where “[t]he fractional occupancies [*f_i_*(*t*)] vary with time, but the individual structure factors [***F***_*i*_(***H***)] associated with each conformation [*i*] do not” (Moffat, 1989). Here, Eq. (1) is modified slightly from the original formulation of Moffat for consistent notations in this review. He also stated that a similar equation holds in real space given the inversion property of the Fourier transform (Moffat, 2001). Therefore,

$$\rho \left(t,r\right)=\sum_{i}f_{i}(t)\rho_{i}(r),$$ (2)

where *ρ*(*t*, ***r***) is the measured electron density at the reaction time *t* and the location ***r*** in the real space of the protein molecule, while *ρ_i_*(***r***) is the electron density contribution from a time-independent, intermediate structure *i* at the same location ***r***. Henderson and Moffat (1971) showed that the measurements of difference Fourier maps Δ*ρ*(*t*, ***r***) are more sensitive to small changes and less prone to systematic errors. In practice, the electron density *ρ* in Eq. (2) is usually replaced by difference electron density Δ*ρ*. A difference Fourier map can be synthesized using a Fourier coefficient set of *F*(*t*, ***H***) – *F*(*t*_reference_, ***H***), where a reference time point is usually chosen as the time point when the ground state can be measured, that is, *t*_reference_ = *t*_0−_. However, any time point before *t* can also serve as a reference such that the difference map reveals the net changes since that reference time point without involving any preceding changes. It is important that the reference dataset *F*(*t*_reference_, ***H***) is collected in the same experiment as *F*(*t*, ***H***). A cross reference collected under a different experimental setting usually causes large systematic errors that would swamp desired signals in the resulting difference map. Proper scaling and weighting are also critical for achieving the best possible quality of difference maps (Ren *et al.*, 2013, 2001; Šrajer *et al.*, 2001; and Ursby and Bourgeois, 1997).

On the left side of Eqs. (1) and (2), ***F***(*t*, ***H***), *ρ*(*t*, ***r***), and Δ*ρ*(*t*, ***r***) are experimental observations that contain mixed signals from the reactants, intermediates, and products, while the functions on the right side are the desired solutions. These structure factor sets ***F***_*i*_(***H***) and their corresponding electron density maps *ρ_i_*(***r***) or Δ*ρ_i_*(***r***) represent the distinct structures involved in the biochemical reaction, but isolated from one another, while their populations *f_i_*(*t*) vary as a function of time during the reaction. A simultaneous solution entails datasets collected at far more time delays than the number of unknowns such that a linear system can be established and solved by overdetermination. In other words, it would be an overinterpretation if each observation at a given delay time leads to a unique structure.

Three important implications from these equations are worth noting. First, although it is desirable to solve the electron density maps for all pure conformational species that rise, peak, and fall as a function of time throughout a chemical reaction, it would be more practical and informative to resolve electron density maps that depict structural events, often occurring in different parts of the protein structure. More than one structural event could concur in a pure conformational species (see example below). Second, the time-dependent function *f_i_*(*t*) for each structural species *i* is shared not only between Eqs. (1) and (2) but also among all reflections ***H*** in reciprocal space and every position ***r*** in real space. These time-dependent functions are, therefore, space-independent. They depict the population rise and fall of reactants, intermediates, and products throughout a chemical reaction. Third, both the structure factor set ***F***_*i*_(***H***) and the electron density map *ρ_i_*(***r***) or Δ*ρ_i_*(***r***) corresponding to the structural species *i* are time-independent spatial functions in reciprocal and real spaces, respectively. Therefore, the first step toward the desired solutions is to factorize time-resolved observations, either ***F***(*t*, ***H***), *ρ*(*t*, ***r***), or preferably Δ*ρ*(*t*, ***r***), into space-independent temporal functions and time-independent spatial functions. We shall demonstrate below how singular value decomposition (SVD) is utilized to achieve this first goal elegantly.

If a reaction under investigation conforms to the Arrhenius behavior, the temporal functions *f_i_*(*t*) can be expressed as a sum of several exponential functions so that a kinetic model of the chemical reaction would be in sight (Moffat, 2001). If all *f_i_*(*t*) functions involve only a single exponential in the form of exp(−*t*/*τ*), it is a strong indication that the reactant converts to the product in a single chemical step of a time constant *τ*, and no intermediate species is detected. It is important to note that two time points *t*_1_ and *t*_2_ do not guarantee a clean resolution of two distinct structural species. When *t*_1_ and *t*_2_ are well separated on both sides of the time constant *τ*, they would carry greater resolving power to differentiate the product from the reactant to a certain extent. When an intermediate state exists due to a rate-limiting step, the functions *f_i_*(*t*) would exhibit a biphasic behavior with two different time constants *τ*_1_ and *τ*_2_, such as *f_i_*(*t*, *τ*_1_, *τ*_2_). In this case, three time points flanking and interleaving with the time constants would be the best choice to resolve three structural species. Regardless of the choice of time points, signals remain heterogeneous at all times unless the distinct reaction steps occur decades apart such that an equilibrium is reached temporarily in the middle of the reaction. In other words, the resolving power of time is limited by an exponential function.

Although not guaranteed, if Eqs. (1) and (2) can be transformed into another form where at least one of the time-dependent functions involves only a single time constant, say *f*_1_(*t*, *τ*_1_), this transformation is said to accomplish a deconvolution of one chemical process from the other. It would be highly informative to figure out what corresponding electron density maps *ρ*_1_(***r***) and *ρ*_2_(***r***) would satisfy the transformation such that at least one of the deconvoluted maps can be visualized as a pure chemical species. This deconvolution after SVD represents the second step in obtaining the desired solutions in Eqs. (1) and (2). We will demonstrate how such deconvolution can be achieved by a rotation after SVD.

In our years working in Moffat lab, we saw little relevance of the Arrhenius behavior of a reaction to our future careers. As our scientific inquiries expanded, we had to find practical solutions to different problems in our hands, which often demanded data under a variety of experimental conditions. We started to veer off track by broadening the definition of the variable *t* and replacing it with a vector of multi-parameter metadata ***X*** = (*t*, *T*, *λ*, pH, …). Hence,

$$\rho \left(X,r\right)=\sum_{i}f_{i}(X)\rho_{i}(r).$$ (3)

Here the functions of metadata *f_i_*(***X***) could span ranges of many physical and chemical parameters beyond time *t*, such as reaction temperature *T*, excitation wavelength *λ* and power, ligand concentration, and buffer pH. For example, studying a particular reaction at different temperatures could reveal unique aspects of the reaction scheme, whether above or below the glass transition temperature (Schmidt *et al.*, 2010; Yang *et al.*, 2011). Deconvolution becomes ever more necessary in such multi-parameter structural study. The purpose of deconvolution also expands far beyond a kinetic model of reaction as illustrated by the case studies and research projects reviewed below. Regardless of the nature of metadata, the property of factorization remains the same for broadly defined dynamic data. In other words, only the population of each structural species *i*, not the conformation itself, depends on the metadata of experimental conditions. If a solution of factorization equivalent to Eq. (3) can be found such that one particular parameter *x* is isolated from the rest, such as

$$\rho \left(X,r\right)=f_{k}\left(x\right)\rho_{k}\left(r\right)+\sum_{i\neq k}f_{i}(X_{\neq x})\rho_{i}(r),$$ (4)

we say that the parameter *x* is successfully deconvoluted or deconvolved from the other metadata ***X***_≠__*x*_, that is, the coefficient *f_k_*(*x*) as a function of the parameter *x* and the corresponding structural species *ρ_k_*(***r***) are determined.

In summary, there are two intertwined aspects of resolving structural heterogeneity in any dynamic studies including time-resolved crystallography: to establish the population change of a structural species as a function of a given parameter *x* in metadata and to resolve structural changes in response to the change of *x* and *x* alone. In this review, we discuss key concepts of SVD in the context of spectroscopic and crystallographic applications and demonstrate how a multi-dimensional rotation enables isolation of chemically sensible structural signals in several case studies and real projects.

## SINGULAR VALUE DECOMPOSITION (SVD)

**FIG. 1.**
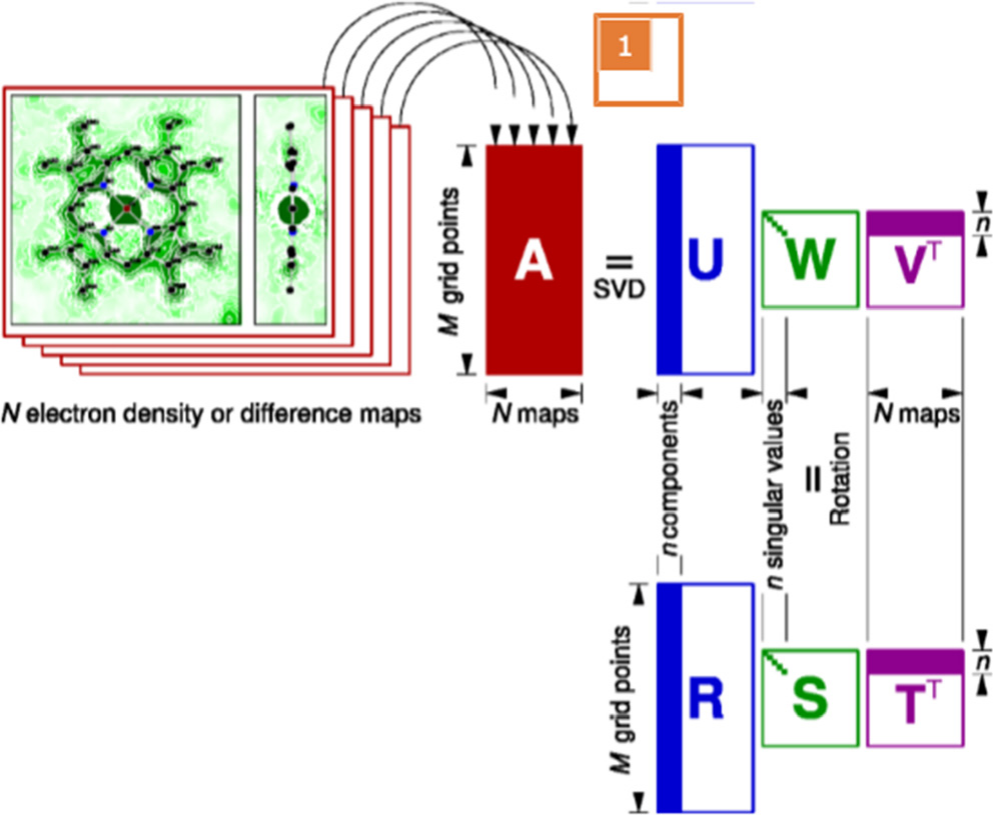
Schematic drawing of singular value decomposition (SVD) and deconvolution. The data matrix **A** consists of the core data where each of *N* electron density maps occupies a column of *M* grid points. The deconvolution scheme involves two steps. In the first step, SVD factorizes matrix **A** into **UWV**^T^. The top *n* singular triplets are considered significant. They are represented by the top *n* components in matrix **U**, the top *n* singular values in matrix **W**, and the top *n* rows in matrix **V**^T^. In the second step, a rotation is performed to find an alternative solution of factorization **RST**^T^. The purpose of this rotation is to obtain new components in matrix **R** that may carry physically or chemically meaningful structural features, at least for some of them.

An electron density map *ρ*(***X***, ***r***), or a difference map Δ*ρ*(***X***, ***r***), consists of an array of density values on grid points within a region of interest. To perform SVD on electron density maps, all *M* grid points in a three-dimensional map can be serialized into a one-dimensional sequence of density values. What serialization protocol to use is not important as long as it is consistently applied to all maps with the same grid setting and size, and a reverse protocol is available to erect a three-dimensional map from a sequence of *M* densities. A set of *N* serialized maps fill the columns of a data matrix **A** with no specific order, so that the width of **A** is *N* columns spanning the space of metadata ***X***, and its length is *M* rows spanning the real space of ***r*** (Fig. 1). Notice that the metadata ***X*** consisting of experimental conditions and the real space coordinates ***r*** are not part of the data matrix **A** for SVD. Only the electron density values are entered in **A** for SVD, hence called the core data, as opposed to the metadata. If a consistent protocol of serialization is used, the corresponding voxels from all *N* maps would occupy a single row of matrix **A**. Such strict correspondence in a given row of matrix **A** is important. Changes of the density values in a row from one map to another could arise from signals, systematic errors, or noises. Although this review mainly concerns electron densities as core data, SVD and its associated analytical procedures are equally applicable to many other types of data, such as spectroscopic data used for case studies below and interatomic distance matrices (Ren, 2016, 2013a, 2013b).

SVD of the data matrix **A** results in $A=\mathrm{UW}V^{T}$, also known as matrix factorization (Fig. 1). Matrix **U** has the same shape as **A**, that is, *N* columns and *M* rows. The *N* columns contain a series of decomposed basis components ***U***_*k*_, known as left singular vectors of *M* items each, where *k* = 1, 2, …, *N*. For crystallographic maps, each component ***U***_*k*_ spanning the real space of ***r*** can be erected using the reverse protocol to form a three-dimensional map. These decomposed elemental maps ***U***_*k*_ can be presented in the same way as the original maps using popular molecular graphics software such as Coot and PyMol. Notice that these decomposed elemental maps or map components ***U***_*k*_ are independent of any metadata. That is to say, these map components remain constant while the metadata vary, which satisfies the dependency requirement of the function *ρ_i_*(***r***) in Eq. (3).

The second matrix **W** is a square matrix that contains all zeros except for *N* non-negative values on its major diagonal, known as singular values *w_k_* (Fig. 1). The magnitude of *w_k_* is considered as the weight or significance of its corresponding component ***U***_*k*_. The third matrix **V** is also a square matrix of *N* × *N*. Each column of **V** or row of its transpose $V^{T}$, known as the right singular vector ***V***_*k*_, contains the relative compositions of ***U***_*k*_ in each of the *N* original maps in **A**. Each right singular vector ***V***_*k*_ can be considered as a function of the metadata, which satisfies the dependency requirement of function *f_i_*(***X***) in Eq. (3). Effectively, SVD separates the constant components independent of the metadata from their compositions that depend on the metadata. Thus, the first goal in solving the desired unknowns as depicted by Eq. (3) is essentially achieved by SVD, which factorizes the experimental observations in **A** into a set of spatial functions independent of metadata and their corresponding population changes as a function of metadata. This achievement is further clarified by reconstitution of several most significant components ***U***_*k*_.

## RECONSTITUTION

A singular triplet denotes (1) a decomposed component ***U***_*k*_, (2) its singular value *w_k_*, and (3) the composition function ***V***_*k*_. Singular triplets are often sorted in descending order of their singular values *w_k_*. Only a small number of *n* significant singular triplets identified by the greatest singular values *w*_1_ through *w_n_* can be used in a linear combination to reconstitute a set of maps that closely resemble the original ones in matrix **A**, where *n* < *N*. For example, the original map $\rho_{j}(X,r)$ in the *j*th column of matrix **A** under a certain experimental condition ***X*** can be closely approximated by the *j*th reconstituted map $\varrho_{j}(X,r)$.

$$\rho_{j}\left(X,r\right)\approx \varrho_{j}\left(X,r\right)=\sum_{k=1}^{n}w_{k}v_{kj}U_{k}=\sum_{k=1}^{n}c_{kj}U_{k},$$ (5)

where *v*_1__*j*_, *v*_2__*j*_, …, *v_nj_* are the first *n* items in the *j*th row of matrix **V**, and *w_k_v_kj_* are denoted as the coefficient set *c_kj_* of the linear combination of ***U***_*k*_. Each component ***U***_*k*_ spanning the real space ***r*** is independent of any metadata, while how much of each component is required for the approximation, that is, *c_kj_*, depends on the metadata [compare Eqs. (3) and (5)]. An analogy in everyday life to this linear combination would be a cooking recipe, with the map components being the ingredients and the corresponding coefficients describing the quantities of the ingredients, except that in a reconstituted map, some coefficients can be negative.

Assuming that the singular values after *w_n_* are markedly smaller than those from *w*_1_ through *w_n_*, the components after ***U***_*n*_ are considered insignificant, thus often excluded in this approximation. That is, the data matrix **A** is said to have a low effective rank of *n*. As a result, the structural information evenly distributed in all *N* original maps is effectively concentrated into a far fewer number of *n* significant components, known as information concentration or dimension reduction. On the other hand, the trailing components in matrix **U** contain inconsistent fluctuations and random noises. Excluding these minor components effectively removes noises (Schmidt *et al.*, 2003). The least squares property of SVD guarantees that the rejected trailing components sum up to the least squares of the discrepancies between the original core data and the approximation using the accepted components.

However, no clear boundary is guaranteed between signals, systematic errors, and noises. Systematic errors could be more significant than the desired signals. Therefore, it is sometimes necessary to exclude certain components from 1 through *n*. If systematic errors are correctly identified, they can be effectively removed by eliminating the corresponding components during reconstitution. Since systematic errors are just unwanted signals, deconvolution of systematic errors is equally important as deconvolution of desired signals. While the desired signals arising from a chemical reaction are expected to follow a kinetic model, a variety of systematic errors usually do not.

## CASE STUDIES OF SVD IN SPECTROSCOPIC DATA ANALYSIS

For better clarity, we opt to use (difference) absorption spectra instead of electron density maps to illustrate the principles of SVD as they are equally applicable to both electron density maps and structure factor sets.

### Dark reversion of bacteriophytochrome

**FIG. 2.**
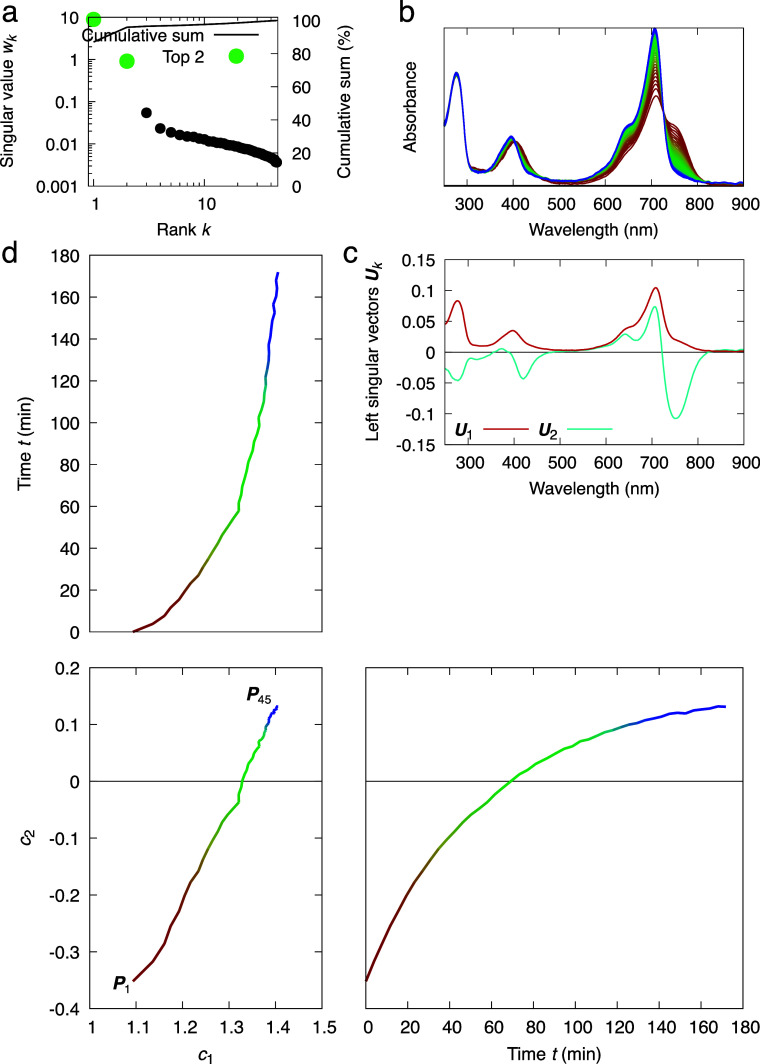
SVD analysis of a time series of absorption spectra following the dark reversion process of bacteriophytochrome in solution. (a) Singular values suggesting two most significant spectral components in this time series. (b) Absorption spectra of a bacteriophytochrome collected in a time series during dark reversion. 45 spectral curves from red to blue are measured in equal time interval spanning a period of three hours. (c) Left singular vectors corresponding to the most significant spectral components. (d) Orthographical projections of the three-dimensional space of *c*_1_, *c*_2_, and time *t*. The coefficients *c*_1_ and *c*_2_ are associated with the top two spectral components, resulting from the top two right singular vectors multiplied by the corresponding singular values *w*_1_ and *w*_2_ as defined in Eq. (5). *c*_1_ and *c*_2_ exhibit a linear correlation while each coefficient follows an exponential function of time *t*. Each point on the linear trace from ***P***_1_ to ***P***_45_ corresponds to one observed absorption spectrum in the time series.

In the first case study, we examine a time series of absorption spectra recorded during the dark reversion process of a full-length bacteriophytochrome *Rp*BphP2 from photosynthetic bacterium *Rhodopseudomonas palestras* [Fig. 2(b)]. *Rp*BphP2 incorporates a linear tetrapyrrole biliverdin as a chromophore, and undergoes reversible photoconversion between a red-light-absorbing Pr state and a far-red-light-absorbing Pfr state (Giraud *et al.*, 2005; Yang *et al.*, 2015). Typical for a canonical bacteriophytochrome, *Rp*BphP2 adopts the Pr state in the dark with *λ*_max_ = 707 nm. Upon red light illumination, it photoconverts to the Pfr state with *λ*_max_ = 748 nm. In the absence of any light, *Rp*BphP2 reverts slowly but spontaneously to the Pr state with a half-time of about 1 h.

A time series of absorption spectra is measured at 45 time points evenly spaced over 3 h during the dark reversion of *Rp*BphP2 in solution. The starting sample shows spectral features characteristic to both Pr and Pfr states following red light exposure at 660 nm [reddest curve in Fig. 2(b)]. The absorbance values of each spectrum cover the wavelength range between 250 and 900 nm occupying a column of the data matrix **A**. SVD of the matrix **A** results in two significant singular values far greater than the rest [Fig. 2(a)]. The corresponding two most significant spectral components ***U***_1_ and ***U***_2_, or the left singular vectors, are shown in Fig. 2(c). The first component ***U***_1_ resembles the dark resting spectrum of Pr but with a small shoulder at the far-red region somewhat like the light spectrum of Pfr. Therefore, ***U***_1_ contains mixed signals from both states. The second component ***U***_2_ features both positive and negative peaks similar to a difference spectrum. When the right singular vectors are plotted against the metadata, here time *t* [Fig. 2(d)], both seem to follow exponential functions. It would be very informative to plot the right singular vectors against each other, revealing the trace of the compositional changes of the corresponding spectral components and facilitating a subsequent rotation for deconvolution of pure spectra.

The dark reversion of *Rp*BphP2 proceeds linearly from a mixture of Pr and Pfr states back to the Pr state without any detectable intermediate species since *c*_1_ correlates with *c*_2_ in a straight line [Fig. 2(d)] suggesting that both right singular vectors follow exponential functions of the same time constant. In other words, only two distinct spectral species of the bilin chromophore are detected. However, this observation does not exclude the possibility of any spectrally silent intermediate conformation. Here, the observed smooth evolution in spectral change simply reflects the continuously varying composition between Pfr and Pr states. It would be incorrect to conclude that the temporal smoothness displayed in the time series of absorption spectra originates from the conformational continuity of the bilin chromophore during its dark reversion. In this case study, it seems easy to understand Moffat's warning of “no temporal coherence” among bacteriophytochrome molecules in solution (Moffat, 1989). Yet, similar erroneous conclusions are not uncommon in the literature of time-resolved crystallography, where each and every electron density map in a time series was usually interpreted and refined as a distinct structure while a collection of such structures are said to be “time-resolved.” In reality, there is no guarantee that any time point would represent a pure state during a reaction or process, including those time points at the beginning and end. Furthermore, a simple SVD procedure does not produce pure spectra of the Pfr and Pr states. As discussed later in the review, deconvolution of pure spectra would entail additional data processing known as a rotation in the most significant subspace identified by SVD. This case study continues after we introduce the method of deconvolution.

### Photoreaction of photolyase

**FIG. 3.**
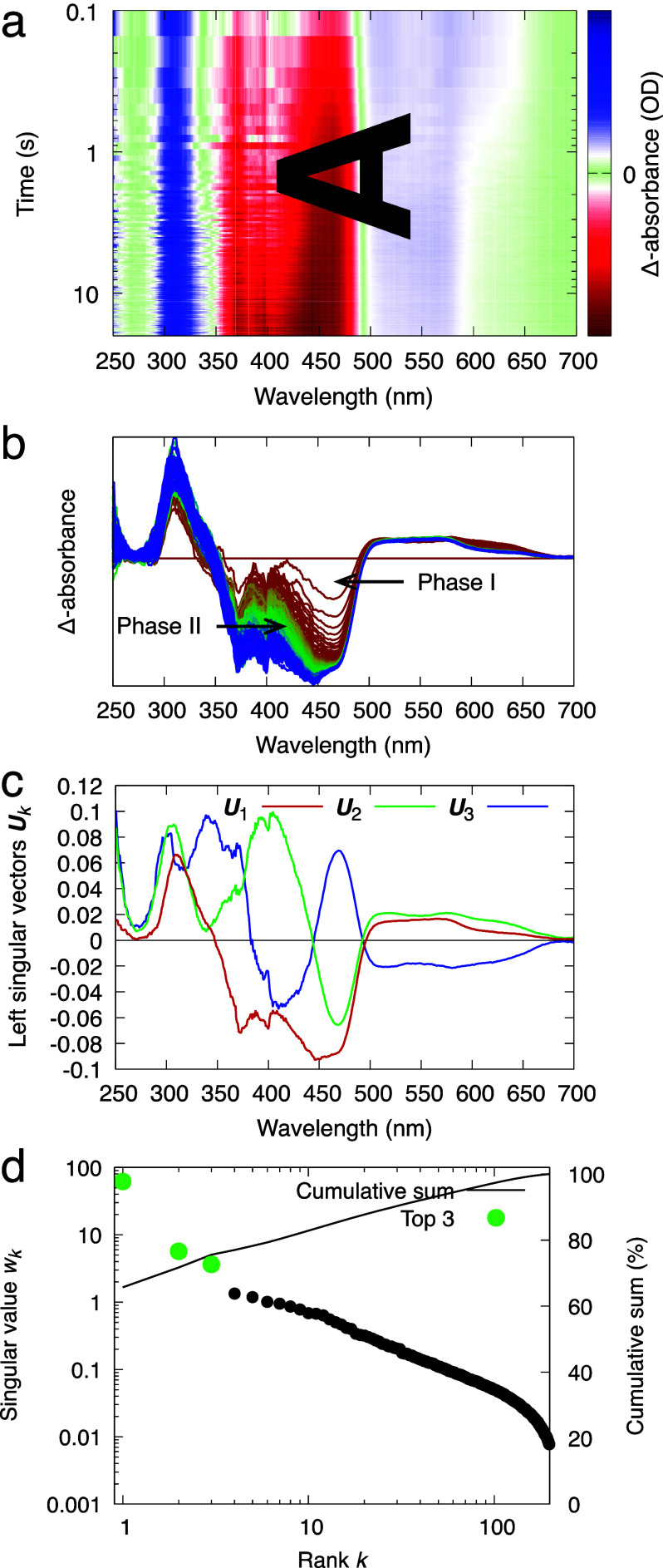
Time series of light-induced difference absorption spectra in photolyase PhrB. The time series is recorded during a period of 20 s under continuous light illumination. Difference spectra are obtained by subtracting the reference absorption spectrum at the dark resting state. (a) The time series of difference spectra are displayed as an image in the two-dimensional space of wavelength and time. This image is essentially the data matrix **A** subjected to SVD. (b) The time series is displayed as difference spectral curves from red to blue in equal time interval. The loss of absorbance in two wavelength regions clearly exhibits two distinct rates as marked phases I and II. (c) Top three spectral components of this time series revealed by SVD. (d) Singular values.

**FIG. 4.**
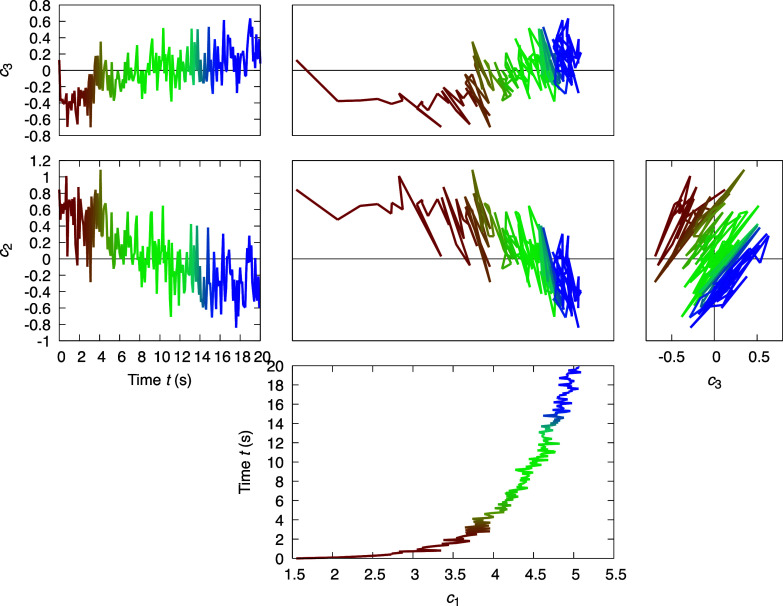
Traces of three SVD coefficients obtained from the time series following the photoreactions in PhrB. The four-dimensional space of *c*_1_, *c*_2_, *c*_3_, time *t* is presented by the orthographical projections. Each pair of adjacent panels, horizontal or vertical, can be folded along a line between them to erect a three-dimensional space. The time series from start to end is colored from red to blue.

In the second case study, we examine a more complicated scenario involving the interplay among three different cofactors of a photolyase PhrB where these redox cofactors, namely, a catalytic flavin cofactor FAD, an auxiliary ribolumazine cofactor DMRL, and an inorganic iron–sulfur cluster ([4Fe4S]), exhibit overlapping spectral features (Graf *et al.*, 2015; Oberpichler *et al.*, 2011; and Ren *et al.*, 2023). This time series of difference absorption spectra is measured from a single PhrB crystal under continuous illumination of violet light at 405 nm [Figs. 3(a) and 3(b)]. SVD of the difference spectra at 200 time points evenly spaced in 20 s produces three outstanding singular triplets [Figs. 3(c) and 3(d)]. The first component ***U***_1_ carries strong signals arising from FAD photoreduction characterized by the negative sharp edge at 485 nm. The other two major components ***U***_2_ and ***U***_3_ show a transition from a decent correlation at short wavelengths to an anticorrelation at long wavelengths [Fig. 3(c)]. The four-dimensional space of *c*_1_, *c*_2_, *c*_3_, and *t* is presented in several orthographical projections in Fig. 4. Only *c*_1_ seems to follow an exponential function, while large fluctuations are observed in the top three right singular vectors. Interestingly, these fluctuations are not random as they exhibit a preferred orientation. Once again, this case study shows that a pure mathematical decomposition alone is insufficient for revealing the chemically meaningful property of the sample. This inadequacy has motivated our efforts in recent years to develop a method of deconvolution to extract pure states.

## THE ORTHONORMAL PROPERTY OF SVD

A solution set of SVD guarantees that the columns in **U** and **V**, namely, the left and right singular vectors ***U***_*k*_ and ***V***_*k*_, are orthonormal. That is, ***U***_*h*_•***U***_*k*_ = ***V***_*h*_•***V***_*k*_ = 0 (ortho) and ***U***_*k*_•***U***_*k*_ = ***V***_*k*_•***V***_*k*_ = 1 (normal), where *h* ≠ *k* but both range from 1 to *N*. The orthonormal property also holds for the row vectors. As a result, each component ***U***_*k*_ is independent of the other components in matrix **U**. In other words, one component cannot be represented by a linear combination of any other components. In dynamic experiments, however, protein structural responses to two physical or chemical parameters in the metadata, such as temperature and pH, are not necessarily orthogonal. They could even exhibit some correlation. Therefore, the decomposed left singular vectors ***U***_*k*_ from the pure mathematical decomposition of a matrix may not necessarily represent any physically or chemically meaningful changes.

Due to the orthonormal property of SVD, an *N*-dimensional Euclidean space is established, and its most significant subspace is defined by the first *n* dimensions. Each coefficient set ***P***_*j*_ = (*c*_1__*j*_, *c*_2__*j*_, …, *c_nj_*) of the *j*th reconstituted map is located in this *n*-dimensional subspace, such as the two-dimensional subspace of *c*_1_ and *c*_2_ in Fig. 2(d) and the three-dimensional subspace of *c*_1_, *c*_2_, and *c*_3_ in Fig. 4. All coefficient sets for *j* = 1, 2, …, *N* used in the reconstitution of the *N* original electron density maps (or spectra) in a least squares sense can be represented by *N* points or vectors ***P***_1_, ***P***_2_, …, ***P***_*N*_ in the Euclidean subspace [Figs. 2(d) and (4)]. This *n*-dimensional subspace essentially defines a conformational space surveyed by the jointly analyzed core data. While right singular vectors are traditionally presented as functions against some selected metadata, such as time *t*, here we emphasize the importance to present right singular vectors scaled by their corresponding singular values, that is, the coefficient set as defined in Eq. (5), as a multi-dimensional scatter plot. Each reconstituted electron density map (or spectrum) and the corresponding observed map are presented as a dot in the conformational space at a position determined by the coefficient set ***P***_*j*_. When the subspace has dimensionality greater than two, the conformational space is presented as multiple two-dimensional orthographical projections (Fig. 4). These scatter plots are highly informative for examining the relationship between the electron density maps and their metadata, and for conducting deconvolution by rotation in this Euclidean subspace.

Given two coefficient sets ***P***_*i*_ ≈ ***P***_*j*_ located close to each other in the conformational space, the corresponding structures *i* and *j* are expected to have similar conformations arising from nearly identical reconstitutions. Conversely, two dots far apart from each other in the conformational space suggest two distinct conformations with dissimilar compositions of the map components. For dynamic data collected in a time series, establishing a trajectory is straightforward given that the time stamp of each data point is known. For a collection of electron density maps without a concrete order, it is also possible to establish a trajectory in this conformational space even when the temporal order of the core data is absent, assuming that population change evolves smoothly along a reaction pathway (Ren, 2016, 2013a, 2013b). The order of structures in a series would inform the causation and consequence of structural changes, which in turn reveals structural mechanism. In addition, an off-trajectory location or those between two clusters of observed structures would reveal rare conformations that have never been experimentally captured likely due to energy barriers. Such hypothetical structures can be very informative when they are refined against reconstituted electron density maps (Ren, 2022) or against reconstituted distance matrices using molecular distance geometry (Ren, 2016, 2013a, 2013b).

## ROTATION IN EUCLIDEAN SUBSPACE OF SVD

The default solution set of SVD usually carries signals of complicated physical and chemical meanings that are hard to discern. Therefore, SVD alone provides no direct answer to “what-does-it-mean.” A basis component ***U***_*k*_ must be interpreted in the context of the metadata. It is important to note that the factorized set of matrices **U**, **W**, and **V** from SVD is not the only solution. The question is: Would alternative solution sets present a physically meaningful interpretation? The idea of rotation following SVD was introduced by Henry and Hofrichter (1992). However, the protocol they put forward does not preserve the orthonormal and least squares properties of SVD. Ren proposed a different rotation protocol that takes metadata into consideration so that the relationship between the core data and metadata can be directly examined (Ren, 2019). This rotation protocol enables a numerical deconvolution of multiple physical and chemical factors following a pure mathematical decomposition, thereby providing a route to answer the question of “what-does-it-mean.” The Ren rotation shall not be confused with a rotation in the three-dimensional real space where molecular structures reside.

To show a clear trend as a function of metadata, it is often necessary to change the perspective via a rotation in the *n*-dimensional Euclidean subspace. It can be demonstrated that this theorem of rotation holds: Two linear combinations are equal before and after the same rotation is applied to both the basis components and their coefficients in a two-dimensional subspace of *h* and *k*. That is,

$$c_{h}U_{h}+c_{k}U_{k}=f_{h}R_{h}+f_{k}R_{k},$$ (6)

where *c_h_* and *c_k_* are the coefficients of the basis components ***U***_*h*_ and ***U***_*k*_ before the rotation, and *f_h_* and *f_k_* are the coefficients of the rotated basis components ***R***_*h*_ and ***R***_*k*_, respectively. The same rotation of an angle *θ* is applied to both the components and their coefficients:

$$\left\{\begin{array}{c} R_{h}=U_{h}\;\text{cos}\;\theta -U_{k}\;\text{sin}\;\theta ,\\ R_{k}=U_{h}\;\text{sin}\;\theta +U_{k}\;\text{cos}\;\theta . \end{array}\right.$$ (7)

Obviously, the rotated components ***R***_*h*_ and ***R***_*k*_ remain mutually orthonormal and orthonormal to other components. And

$$\left\{\begin{array}{l} f_{h}=s_{h}t_{h}=c_{h}\;\text{cos}\;\theta -c_{k}\;\text{sin}\;\theta ,\\ f_{k}=s_{k}t_{k}{=c}_{h}\;\text{sin}\;\theta +c_{k}\;\text{cos}\;\theta . \end{array}\right.$$ (8)

Here $s_{h|k}=\sqrt{\sum f_{h|k}^{2}}$ are the singular values replacing *w_h_* and *w_k_*, respectively, after the rotation. As they differ from the original singular values, the descending order of the singular values no longer holds. With the rotation, ***T***_*h*__|__*k*_ = (*t_h_*_|__*k*__1_, *t_h_*_|__*k*__2_, …, *t_h_*_|__*kN*_) = (*f_h_*_|__*k*__1_, *f_h_*_|__*k*__2_, …, *f_h_*_|__*kN*_)/*s_h_*_|__*k*_ are new right singular vectors replacing ***V***_*h*_ and ***V***_*k*_, respectively. ***T***_*h*_ and ***T***_*k*_ remain mutually orthonormal and orthonormal to other right singular vectors that are not involved in the rotation.

This theorem holds because the dot product of two vectors remains unchanged when both vectors rotate by the same angle [Eq. (6)]. It would be straightforward to prove Eq. (6) simply by combining Eqs. (7) and (8) into Eq. (6) and expanding them. All cross terms of sine and cosine would be self-canceled (Ren *et al.*, 2024).

A rotation in two-dimensional subspace of *h* and *k* has no effect on other dimensions, as guaranteed by the orthonormal property of SVD. Furthermore, a multi-dimensional rotation can be achieved by consecutive steps of rotations in many two-dimensional subspaces. The resulting solution set shall retain the orthonormal property of SVD and have no effect on how the core data of protein structures are compared. By transforming one solution set $A=\mathrm{UW}V^{T}$ to an alternative solution set $A=\mathrm{RS}T^{T}$, one may find an appropriate perspective that better elucidates the relationship between the core data and metadata in a clear and concise manner (Fig. 1). The theorem of rotation itself does not mention anything related to deconvolution. However, such rotation enables deconvolution by isolating a specific parameter in the metadata from the others.

After SVD, the structural changes in response to the metadata ***X*** are described by a collection of points ***P***_*j*_ for *N* observations in the most significant Euclidean subspace. Following rotation, if the distribution of ***P***_*j*_ is reoriented along a single dimension *k* as one physical or chemical parameter *x* in the metadata varies but not involving other dimensions, we could convincingly infer that the left singular vector ***R***_*k*_ of this dimension depicts the structural impact of this parameter *x*. Then, Eq. (5) can be revised as

$$\rho_{j}\left(X,r\right)\approx \varrho_{j}\left(X,r\right)=f_{kj}{\left(x\right)R}_{k}+\sum_{i\neq k}f_{ij}\left(X_{\neq x}\right)R_{i}.$$ (9)

Hence, the physical or chemical parameter *x* is deconvoluted from the rest of ***X*** [compare Eqs. (4) and (9)]. Without rotation, the same parameter *x* may involve structural variations along several dimensions that would be too complicated to interpret. If structural changes induced by a parameter *x* cannot be described by a single component ***R***_*k*_, the goal of deconvolution is then to find the minimal dimensionality necessary for describing structural changes in response to *x* (see example below). Would a proper rotation establish a one-on-one correspondence from all physical or chemical parameters to all dimensions? It depends on whether structural changes induced by these parameters are independent of or correlated with one another. If these structural changes are indeed orthogonal, it should be possible to find a proper rotation to cleanly separate them into different dimensions. Otherwise, to deconvolute or deconvolve two correlated changes, it is necessary to perform two rotations, but one at a time (see example below).

If two clusters emerge in the conformational space by examining the distribution of the coefficient set ***P***_*j*_, it is desirable to perform a rotation such that these clusters are aligned along a single dimension *k* without involving other dimensions. As a result, the component ***R***_*k*_ would clearly reveal the structural transition from one cluster to the other. A deterministic solution entails a clear relationship between the core data and metadata. In most cases, a proper rotation is achieved via trial-and-error guided by iterative interpretation of the deconvoluted maps in the context of the subject matter. Although a “bad” rotation may not improve data interpretability, it shall not alter the distribution of the observed data and the shape of the reaction trajectory, because a rotation would not introduce nonexistent features nor eliminate any authentic signals. For example, a rotation cannot alter the observation of a large gap between two clusters of core datasets, although it may make these clusters less obvious from that ill-conceived viewpoint. If a fast chemical step follows a slow one, the short-lived intermediate species may not accumulate enough for detection as it is being produced too slowly in the step leading to it. In this scenario, the proposed rotation would not help because it cannot generate signals not captured in the experimental data. However, if weak signals from a short-lived species are indeed present, their clarity can be significantly enhanced after separation from other variations by a proper rotation.

## CASE STUDIES OF DECONVOLUTION IN SPECTROSCOPIC DATA ANALYSIS

We continue to use the aforementioned case studies to demonstrate the analytical scheme of deconvolution.

### Dark reversion of bacteriophytochrome

**FIG. 5.**
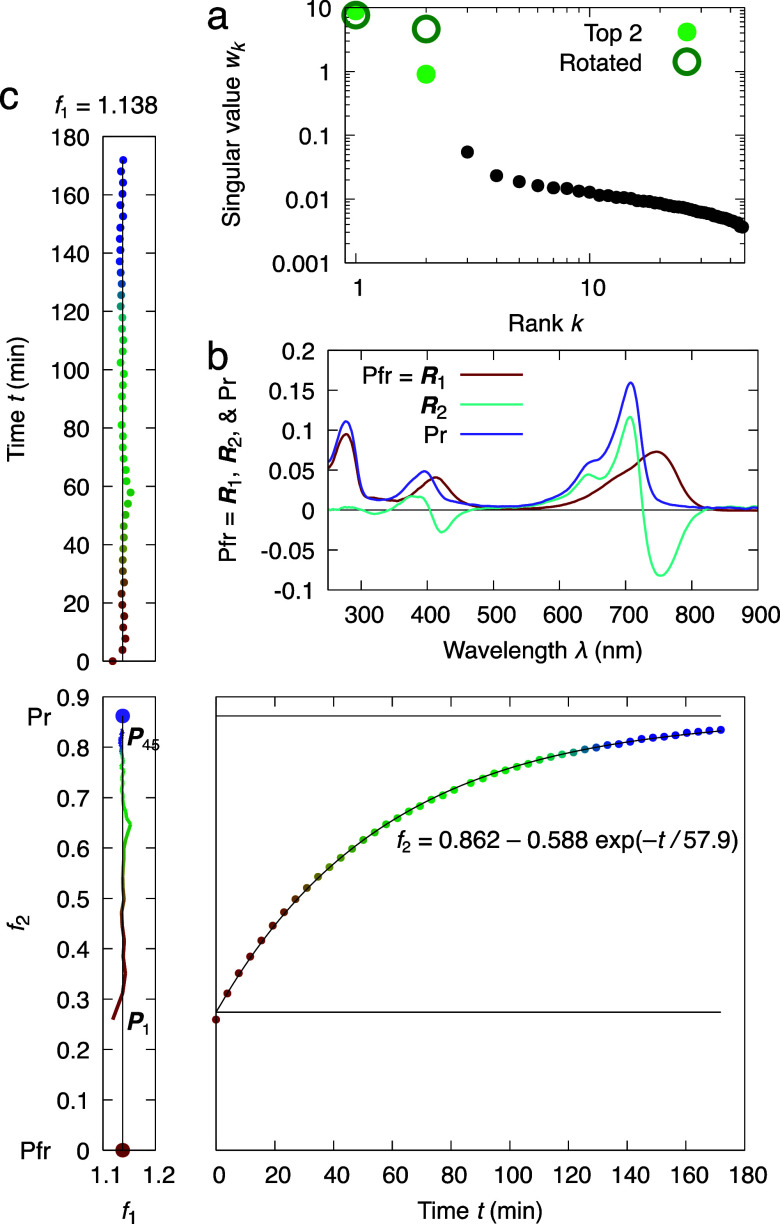
Deconvolution of the pure Pr and Pfr spectra from a spectral time series of dark reversion in bacteriophytochrome. (a) Singular values before and after a rotation in the top two-dimensional subspace. (b) Top two spectral components after rotation. ***R***_1_ depicts the absorption spectrum of the pure Pfr state. The absorption spectrum of the pure Pr state (purple curve) is obtained via a slight extrapolation using a linear combination of 1.138***R***_1_ + 0.862***R***_2_ (purple dot in c). (c) Orthographical projections of the three-dimensional space of *f*_1_, *f*_2_, and time *t* after the rotation. After rotation, the straight-line correlation between *f*_1_ and *f*_2_ becomes perfectly vertical. Here, *f*_1_ is a constant, and *f*_2_ can be fit with a single exponential function *f*_2_ = *b* + *a*[1 − exp(–*t*/*τ*)] with a time constant *τ* = 58 min. The baseline *b* = 0.274 intercepts nicely at the beginning of the time series. Hence, the pure Pfr state corresponds to an extrapolated point marked by the red dot on the vertical line with *f*_1_ = 1.138 and *f*_2_ = 0. The pure Pr state corresponds to the purple dot on the vertical line with *f*_1_ = 1.138 and *f*_2_ = 0.862. Compare to Fig. 2(d).

In this case study, SVD alone performed on the time series of dark reversion does not yield the pure spectra of the Pfr and Pr states in bacteriophytochrome [Fig. 2(c)]. However, if we apply a counterclockwise rotation of 30.9° in the subspace of the top two dimensions where both *c*_1_, *c*_2_, and ***U***_1_, ***U***_2_ are rotated the same way according to Eqs. (7) and (8), the linear combination of these components remains unchanged according to the theorem of rotation [Eq. (6)]. After rotation, the first left singular vector ***R***_1_ reveals the absorption spectrum of a pure Pfr state, while the second ***R***_2_ represents the difference spectrum of Pr–Pfr [Fig. 5(b)]. The straight-line correlation between *c*_1_ and *c*_2_ becomes perfectly vertical, which is how the rotation angle of 30.9° was determined. Therefore, *f*_1_ is a constant, and *f*_2_ can be fit with a single exponential function with a time constant *τ* = 58 min [Fig. 5(c)]. Hence, the pure Pfr state can be derived from an extrapolated point as marked by the red dot on the vertical line. This suggests that the dark reversion experiment did not start with a pure Pfr state; rather the initial sample contained a mixture about 1/3 of Pr and 2/3 of Pfr [reddest curve in Fig. 2(b)]. Similarly, the spectrum of the pure Pr state can be obtained by a slight extrapolation forward beyond the recorded time series at the purple dot in the subspace of *f*_1_ and *f*_2_ [purple curve in Fig. 5(b)]. As a result of rotation, we are able to achieve deconvolution and obtain the pure spectra for both Pfr and Pr states from the heterogeneous data collected throughout the dark reversion process. We intend to use this minimal case study to illustrate what Moffat had envisioned for time-resolved studies. “Time-resolved experimental approaches … attempt to resolve this time-dependent heterogeneity into the structures of homogeneous intermediates during the subsequent data analysis process” (Moffat, 2001).

We may refer the vertical straight line in the subspace of *f*_1_ and *f*_2_ as the reaction trajectory of dark reversion from Pfr to Pr, although only the later 2/3 has been experimentally observed [Fig. 5(c)]. It is noteworthy that this reaction trajectory is an invariant, including both the experimentally captured portion by time-resolved absorption spectroscopy and the extrapolated wings beyond the time series, whether such a straight line is vertical along a single dimension of *f*_2_ after rotation [Fig. 5(c)] or inclined in the original subspace of *c*_1_ and *c*_2_ [Fig. 2(d)]. As implied by the theorem of rotation [Eq. (6)], such invariant reaction trajectory depicts the intrinsic absorption property of this bacteriophytochrome protein regardless of our choice of principal axes. In other words, an alternative coordinate system can be chosen as wish to find a viewpoint of greater clarity and conciseness in data interpretation without altering the invariant reaction trajectory.

### Photoreaction of photolyase

**FIG. 6.**
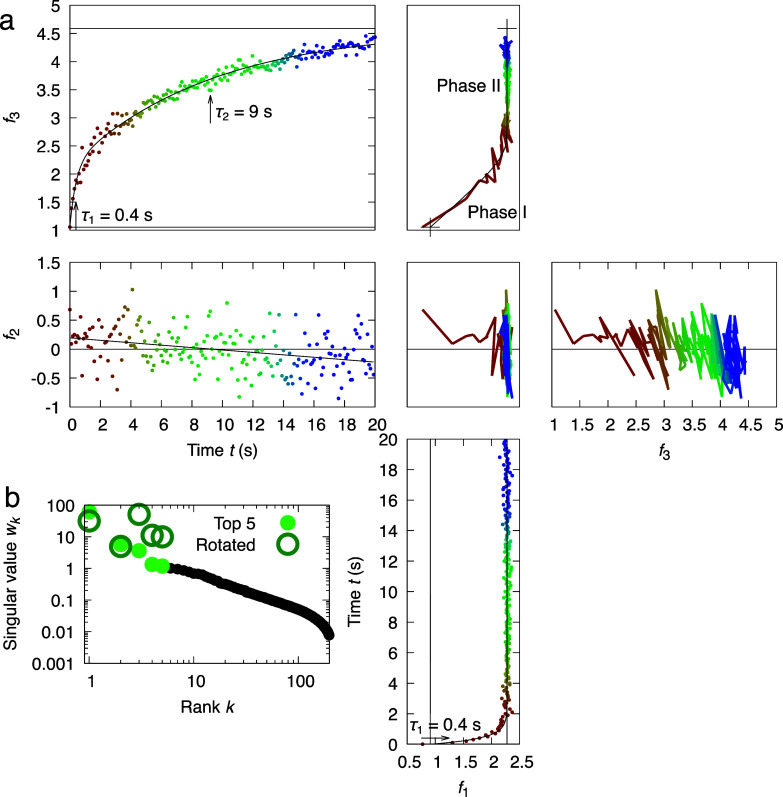
Rotation 1 in top three-dimensional subspace of PhrB photoreaction. (a) Orthographical projections of the four-dimensional space of *f*_1_, *f*_2_, *f*_3_, and time *t* after rotation 1. The time series is colored from red to blue. Two crosses (+) in the top-right panel mark the beginning and end of the photoreaction beyond the recorded time points. (b) Singular values before and after rotation 1.

**FIG. 7.**
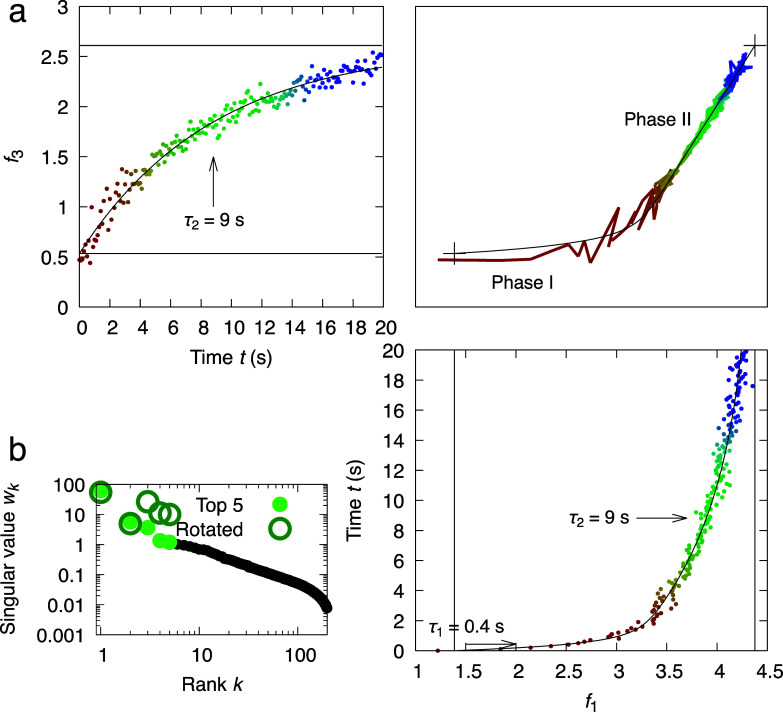
Rotation 2 in top three-dimensional subspace of PhrB photoreaction. (a) The three-dimensional space of *f*_1_, *f*_3_, and time *t* after rotation 2 is presented by the orthographical projections. Notice that the second dimension is omitted since it is the same as in rotation 1 (Fig. 6). The time series is colored from red to blue. Two crosses (+) in the top-right panel mark the beginning and end of the photoreaction beyond the recorded time points. (b) Singular values before and after rotation 2.

**FIG. 8.**
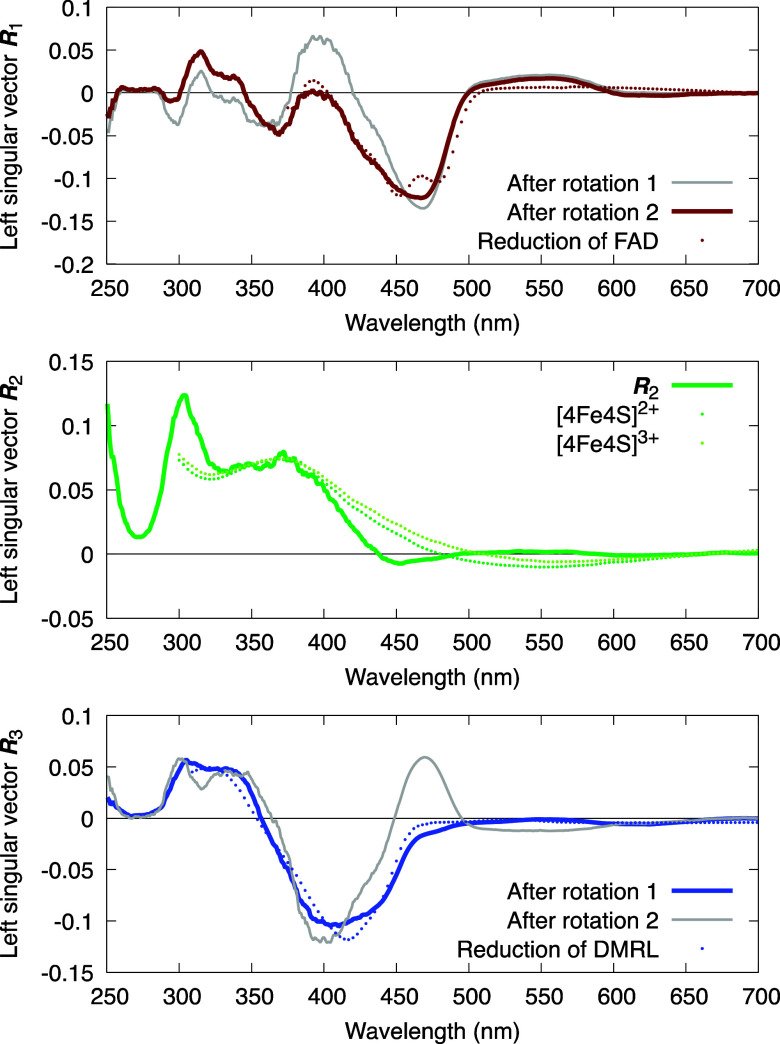
Spectral components of PhrB photoreaction after two different rotations. The dotted lines show experimentally measured spectra from three different proteins, each contains a single cofactor of flavin, [4Fe4S] cluster, or ribolumazine. Overlays of the experimentally measured spectra with the deconvoluted spectral components support that those curves in solid colors represent spectral changes caused by a single cofactor while the gray curves display mixed signals of spectral changes. Such direct comparisons provide cross-validation for the deconvolution scheme based on SVD.

The second case study showcases a multistep rotation performed in the top five-dimensional subspace. After rotation, it becomes clear that the photoreaction of PhrB under violet light is a biphasic process evidenced by the curved correlation between *f*_1_ and *f*_3_. By aligning the slower phase II along the third dimension, *f*_1_ can be fit nicely with a single exponential function of a time constant *τ*_1_ = 0.4 s, while *f*_3_ must be described by a two-exponential function with an additional time constant *τ*_2_ = 9 s [Fig. 6(a)]. This rotation is achieved by a least squares fitting that determines a first step of −43.8° rotation in the subspace of dimensions 2 and 3 followed by a second step of 54.1° rotation in the subspace of dimensions 1 and 3 with several additional steps involving the dimensions 4 and 5. Alternatively, the faster phase I can be aligned to the first dimension so that *f*_3_ can be fit with a single exponential of *τ*_2_ = 9 s, while *f*_1_ requires both exponentials of *τ*_1_ and *τ*_2_ (Fig. 7). These two rotations are off by 32.7° in the subspace of dimensions 1 and 3. The pure difference spectra arising from the photoreduction reactions of FAD and DMRL can be deconvoluted from each other in two alternative rotations but not at the same time. The first spectral component ***R***_1_ after rotation 1 remains as a mixture, while the third component ***R***_3_ depicts photoreduction of the DMRL cofactor alone (Fig. 8). On the other hand, ***R***_1_ represents the change only caused by the photoreduction of FAD, and ***R***_3_ becomes a mixture after rotation 2 (Fig. 8).

This case study underscores an important principle. If two processes are intrinsically interdependent, or if two spectroscopic or structural responses are partially correlated with, instead of orthogonal to, each other, it is impossible to achieve a clean separation in the same Euclidean space established by SVD. The best achievable solution is to use two different rotations to isolate one of the right singular vectors as a single exponential that describes a single chemical process, here *f*_1_(*t*, *τ*_1_) after rotation 1 [Fig. 6(a)] and *f*_3_(*t*, *τ*_2_) after rotation 2 (Fig. 7). Similarly, only one of the left singular vectors at a time is deconvoluted as a pure component attributed to one single process (Fig. 8). A pure component depicting changes caused by a single reaction step cannot coexist with a pure time course of a single exponential. If they do, this process is independent of, hence orthogonal to the others. In other words, a thorough deconvolution as we formulate in Eq. (9) is not always possible.

The significant fluctuations observed in the difference spectra of PhrB are reoriented into the second dimension so that large fluctuations displayed in *f*_2_ are isolated from other dimensions (Fig. 6). ***R***_2_, the fluctuating component, captures spectral changes only in shorter wavelengths <450 nm, largely matching the absorption range of the iron–sulfur cluster [Fig. 8; see also raw difference spectra in Figs. 3(a) and 3(b)], suggesting that the inorganic cofactor acts as a redox capacitor in the photolyase PhrB. This finding alluded to an intriguing proposal that the iron–sulfur cluster could play a protective role in redox active proteins by smoothing out electronic potential spikes arising from an imbalance of redox reactions on the fly, given its large capacity of four electrons and five valence states (Ren *et al.*, 2024, 2023).

## DECONVOLUTION OF ELECTRON DENSITY MAPS

Compared to the case studies in spectroscopy, SVD-based deconvolution is far more complicated when it is applied to a time series of electron density maps or more broadly to a collection of maps obtained under different experimental conditions. The challenge for SVD of crystallographic data is twofold. First, the number of datasets or maps is inevitably limited, that is, the columns of the data matrix **A**. Second, the maps obtained from dynamic crystallography experiments often suffer from significant systematic errors. To address the first challenge, we proposed to use alternative reference datasets so that more difference electron density maps can be produced for SVD analysis (see above). In addition, including electron density maps related by non-crystallographic symmetry (NCS) would help increase the total number of independently measured maps. On the other hand, NCS usually introduces an additional source of systematic discrepancy due to crystal packing. Since deconvolution enables a clean separation of both signals and systematic errors, the benefits gained from SVD of a larger collection of maps, such as better signal to noise ratio and improved interpretability, outweigh additional dimensionality required. In this section, we highlight a few recent findings from SVD-based deconvolution. It is important to point out that other than electron densities, this methodology has also been applied to other types of structural data such as interatomic distance matrices that enabled a large-scale meta-analysis of related structures in the Protein Data Bank (Ren, 2016, 2013a, 2013b).

### Low-frequency oscillations in protein structure

The advent of x-ray free electron lasers (XFELs) has triggered a second wave of time-resolved crystallography in the last decade. By taking advantage of the intense and ultrashort x-ray pulses, dynamic structural events can be captured before the destruction power of x-rays takes effect (Neutze, 2014). When combined with femtosecond laser pulses as a reaction pump, the achievable and highly desirable time resolution is approaching the XFEL pulse duration. Such greatly improved time resolution of x-ray diffraction has enabled direct observation of low frequency oscillations in protein structures, which have been previously detected by ultrafast spectroscopic methods (Perticaroli *et al.*, 2015; Xu and Havenith, 2015). Here low frequencies refer to tens to low hundreds wavenumbers or <10 THz, typically 25–45 cm^−1^ or a period around 1 ps, compared to much higher frequencies of bond stretching and wagging observed by vibrational spectroscopy. These low-frequency signals detected from heme proteins have been attributed to the ligand photodissociation reaction crossing a discontinuity at the intersection of two energy landscapes of the photoexcited state and the ligand dissociated product (Champion, 2005; Gruia *et al.*, 2008; and Rosca *et al.*, 2002). As such low-frequency oscillations are usually damped rapidly after a few cycles, they are often explained as vibrational energy relaxation mainly through the solvent in the protein matrix (Fujisaki and Straub, 2005; Okazaki *et al.*, 2001). Since these spectroscopic signals and theoretical simulations did not offer any molecular image of oscillating structural element, the molecular basis and the functional role of these low-frequency oscillations remain highly speculative.

We applied the SVD-based deconvolution to a time series of XFEL datasets collected by Barends *et al.* (2015) to probe the structural events in carbonmonoxy myoglobin (MbCO) triggered by the photodissociation of the CO ligand. We were able to isolate a near-perfect oscillation that is distinct from the characteristic signals commonly observed by static and time-resolved crystallography, such as the docked CO ligand, the out-of-plane motion of iron, and the heme doming (Ren, 2019). We extracted oscillatory signals for ∼2/3 of a full period, which appears within ∼30 fs upon photolysis and diminishes by the time point of 3 ps. Two major components are required to depict this oscillation and a least squares fitting results in a low-frequency model of ∼40 cm^−1^ or 1.2 THz. Such temporal dependency obtained from time-resolved crystallographic data agrees perfectly with the low-frequency oscillations detected by vibrational coherence spectroscopy (Gruia *et al.*, 2008; Rosca *et al.*, 2002). An immediate question would be “what is oscillating?” The definitive answer resides in the electron density component maps of the left singular vectors in the two-dimensional subspace. In contrast to other components showing excellent correlation with specific structural elements and describing the characteristic signals of photolysis from MbCO, large difference densities manifested in the two-dimensional subspace of oscillation consistently show no association with the heme, ligand, nor protein (see Fig. 7 of Ren, 2019). This suggests that the low-frequency oscillation associates largely with solvent channels. The oscillatory period of 0.83 ps equivalent to ∼40 cm^−1^ is the time that a mechanical wave traverses the molecular dimension. We thus postulate that the low-frequency oscillation arises from a shock wave triggered by femtosecond laser pulse. The shock wave may originate at the heme center, propagates mainly in the solvent channels, then bounces off the protein boundary for a few times, before it completely dissipates. Not surprisingly, the oscillation frequency depends on the size of a protein. It was reported that a slightly smaller heme protein of cytochrome c (cyt c) of 12 kDa and a much larger heme protein of cystathionine *β*-synthase (CBS) of 63 kDa exhibit their dominant low frequencies of 44 and 25 cm^−1^ or periods of 0.76 and 1.3 ps, respectively (Karunakaran *et al.*, 2014, 2010). Given that these heme proteins are nearly globular in shape, the ratios between the cubic roots of their molecular weights could be used to assess their relative linear sizes, that is, cyt c:MbCO:CBS = 0.89:1:1.55, which are highly comparable to the ratios between the oscillatory periods as cyt c:MbCO:CBS = 0.91:1:1.6. This remarkable agreement has lent further support to our proposal. It is important to point out that such structural revelation for vibrational energy relaxation is only made possible by proper deconvolution of the heterogeneous XFEL datasets that record many concurrent structural events.

**FIG. 9.**
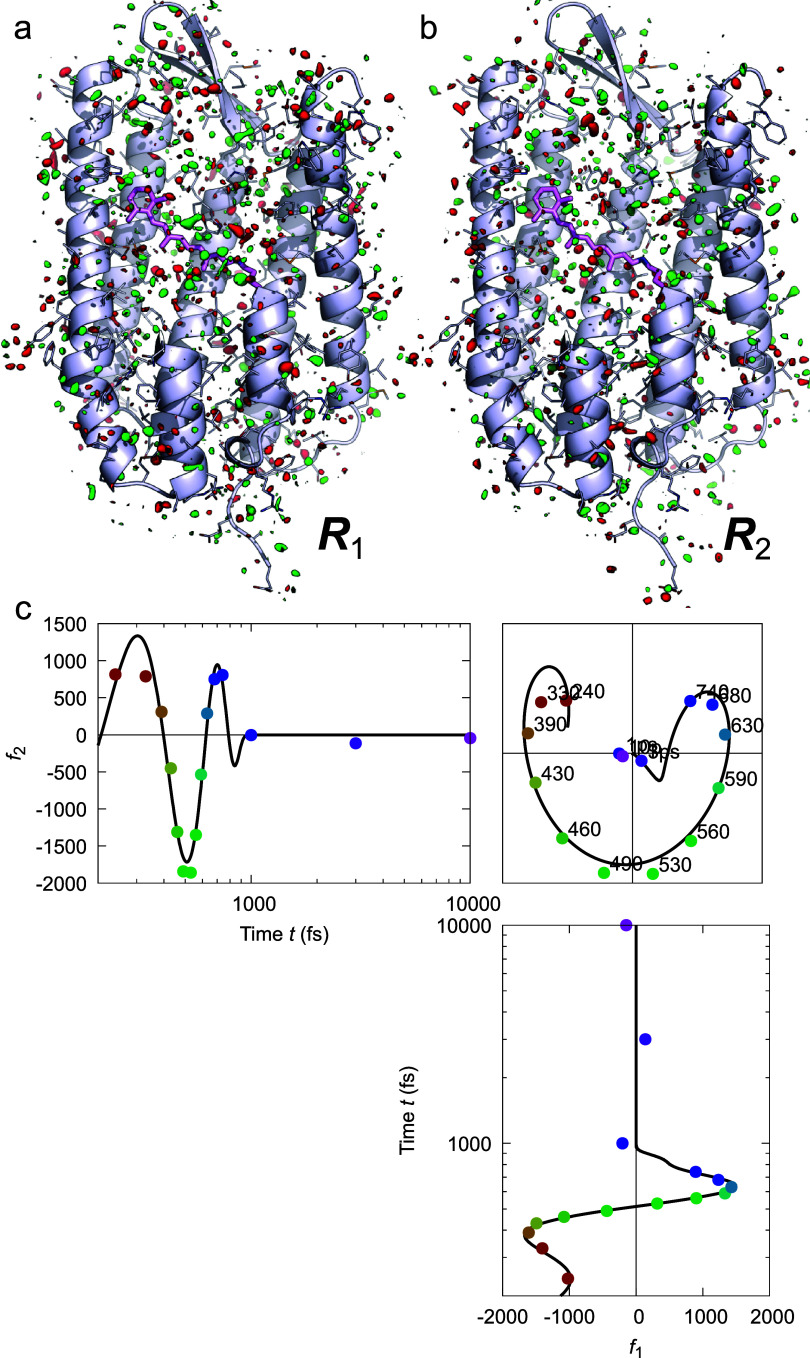
Most significant oscillating components in bacteriorhodopsin (bR). (a) and (b) Two most significant component maps ***R***_1_ and ***R***_2_ after rotation. The difference maps are contoured at ±3*σ* in green and red, respectively. The bR protein is rendered in ribbon model with side chains in thin sticks. The retinal chromophore and its lysine anchor are in thick purple stick model. Parts of the helices are omitted to expose more interior. These oscillating component maps exhibit difference densities largely in the solvent with no significant feature associated with any structural element of the protein in contrast to another non-oscillating component map ***R***_10_ [Fig. 10(a)]. (c) Orthographical projections of the three-dimensional space of *f*_1_, *f*_2_, and time *t*. The time series of datasets are marked in the three-dimensional space as colored dots from red to purple. The coefficients *f*_1_ and *f*_2_ corresponding to the top two component maps after rotation are least squares fitted by a damping sinusoidal function

**FIG. 10.**
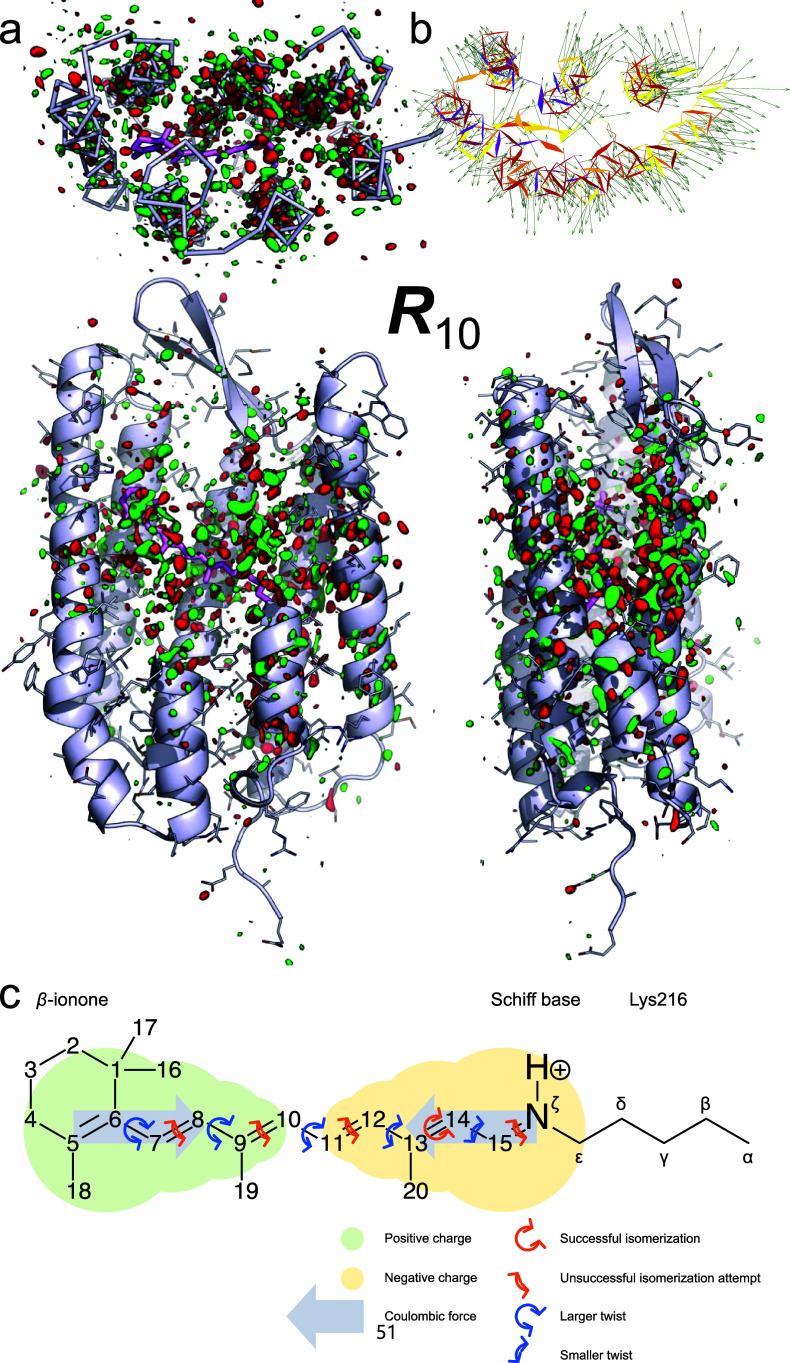
Retinal isomerization sampling in bacteriorhodopsin (bR). (a) Orthographical projections of the component map ***R***_10_ after rotation. The difference map is contoured at ±3*σ* in green and red, respectively. The bR protein is rendered in ribbon and stick models. The retinal chromophore and its lysine anchor are in thick purple stick model. Parts of the structure are omitted to expose more interior. Strong density features are associated with all seven transmembrane helices (top view). The strongest features surround but do not sit on the retinal chromophore. The magnitude of density features reduces toward both ends of the structure (face and side views). This component map depicts an overall expansion of the retinal binding pocket mainly at the waist of the transmembrane helices, captured before the photoisomerization. The negation of the component depicts a contraction of the retinal binding pocket due to recoiling after photoisomerization. (b) Retinal binding pocket expansion. Each arrow starting from an atom points toward the direction of its motion in the I state immediately before isomerization. The length of each arrow is ten times of the actual motion. These arrows collectively show the expansion of the retinal binding pocket. (c) Schematic drawing of a retinal chromophore covalently attached to Lys216 and its isomerization sampling upon light induced charge separation. Under the Coulombic attraction forces, all double bonds along the polyene chain attempt and fail to isomerize except C_13_=C_14_. All single bonds along the polyene chain attempt to rotate. Those single bonds closer to the *β*-ionone ring are less restricted by the protein matrix thereby achieving greater torsional changes. Both isomerization attempts of the double bonds and single bond twists are the driving forces that cause the observed expansion of the retinal binding pocket displayed in (a) and (b).

Low-frequency vibrational dynamics had also been observed from bacteriorhodopsin (bR) in purple membrane and lipid nanodiscs (Ferrand *et al.*, 1993; Johnson *et al.*, 2014). Femtosecond pump-probe spectroscopy study on visual rhodopsin revealed a characteristic frequency at 60 cm^−1^ or 1.8 THz, which led to a premature claim that “the primary step in vision is a vibrationally coherent process” (Wang *et al.*, 1994). In a recent XFEL study on bR, Kovacs *et al.* (2019) collected time-resolved datasets up to 10 ps, from which they reported oscillatory behaviors of torsion angles and interatomic distances around 100 cm^−1^ in the protein and its retinal chromophore. When we reexamined their datasets using the SVD-based deconvolution method and conducted a rotation in a 17-dimensional subspace, we found no signal that can be interpreted as photoisomerization of the retinal. Instead, we obtained extensive oscillatory signals in their data occupying ten significant SVD components, among which five frequencies were isolated from one another and from other types of signals (Ren, 2022). The dominant lowest frequency of 61 ± 2 cm^−1^ matches perfectly with the oscillation reported for visual rhodopsin (Wang *et al.*, 1994). These strong oscillatory signals extracted in the most significant two-dimensional subspace are further modeled by a rapidly damping sinusoidal function that lasts for nearly two periods (Fig. 9). The other four oscillations range from 150 to 400 cm^−1^ (Figs. S3 and S4 of Ren, 2022). These two-dimensional oscillations are good examples to illustrate the concept of minimal dimensionality when a single dimension is not sufficient to describe a process [Eq. (9)]. Despite the excellent sinusoidal signals, the corresponding electron density features show no association with any specific structural element, such as the transmembrane helices or the chromophore [compare Figs. 9(a) and 9(b) to 10(a)], similar to the oscillating components of MbCO. Our findings corroborate the notion that the bR protein was pumped into some higher potential energy surfaces after multiphoton absorption by short pump pulses of excessive peak power density and the bR protein may have entered nonproductive pathways that evade the photoisomerization of the retinal, hence irrelevant to its biological function (Miller *et al.*, 2020). As the oscillating components show, such vibrational energy was oscillating mainly in the solvent before dissipation [Figs. 9(a) and 9(b)]. Twice the wavelength of the shock wave is equivalent to the thickness of the seven-helix bundle of 17 Å given a wave velocity of 1.5 km/s or nm/ps. Here is a critical question: Does the oscillation at the same frequency of 60 cm^−1^ detected from visual rhodopsin arise from the primary step of vision, or a side effect of the retinal photoisomerization, or is it merely an artifact induced by femtosecond pump pulses of excessive peak power density? Key evidence has yet to show whether the oscillation persists after the rhodopsin molecule absorbs a single photon.

$f(t)=[a\text{cos}\left(\omega t-\alpha \right)+ct+b]\text{exp}\left[-{\left(t/\tau \right)}^{\varepsilon }\right].$Two functions *f*_1_(*t*) and *f*_2_(*t*) are well fitted with the common angular frequency *ω* and the damping parameters *τ* and *ε*. As a result of fitting, the entire oscillation can be described as linear combination of *f*_1_(*t*)***R***_1_ + *f*_2_(*t*)***R***_2_.

### Photoisomerization of double bond and isomerization sampling

Light-triggered double bond isomerization is a primary photochemical event in many rearrangement reactions. Understanding this process at the molecular and electronic levels has been a central topic for many time-resolved projects based on synchrotron Laue diffraction and femtosecond serial crystallography at XFELs. Two model systems have been extensively studied to examine the *trans*-to-*cis* photoisomerization of a specific C=C double bond at near atomic resolution. They are the blue light photoreceptor photoactive yellow protein (PYP) incorporated with *p*-coumaric acid chromophore (Genick *et al.*, 1997; Jung *et al.*, 2013; Pande *et al.*, 2016; Perman *et al.*, 1998; and Schotte *et al.*, 2012) and the light-driven proton pump bacteriorhodopsin (bR) mentioned above (Kovacs *et al.*, 2019; Nango *et al.*, 2016; and Nogly *et al.*, 2018). In earlier studies that achieved time resolution of nanoseconds or longer, mixed signals from *trans*/*cis* configurations were not a major concern as the photoisomerization was observed at its completion. However, as more recent studies reached time resolution of picosecond and femtosecond at XFELs, the possibility of structural heterogeneity at early time points during photoisomerization and other photoreactions can no longer be ignored.

On PYP, Jung *et al.* (2013) presented an early intermediate with the double bond C_2_=C_3_ of the chromophore twisted at 80°. An equivalent intermediate refined by Schotte *et al.* (2012) showed a more convincing torsion angle of 33°. Pande *et al.* (2016) reported the *trans*-to-*cis* transition from a torsion angle of 150° to 50° captured around 600 fs. On bR, Nogly *et al.* (2018) described “the trajectory of retinal isomerization” as the C_13_=C_14_ double bond starts to twist from *trans* configuration to a torsion angle of 135° at early hundreds femtosecond, then evolves to 82° at mid hundreds femtosecond, and remains in a distorted *cis* conformation of 37° at 10 ps. Kovacs *et al.* (2019) even tabulated the torsion angles of C_13_=C_14_ double bond at many time points before 10 ps and plotted its continuous evolution against the delay time. While some non-ideal torsion angles seem inevitable in structural refinement due to challenges in thorough deconvolution of *trans* and *cis* signals from each other, the overall picture of PYP focused on the *trans*-to-*cis* transition. However, the interpretation of photoisomerization event as continuous evolution around a double bond is fundamentally flawed.

Here a misconception likely arises from a literal understanding of the phrase “time-resolved crystallography” that disregards the resolving power of time, leading to an overinterpretation of data where every dataset at a given time point gives rise to a uniquely refined conformation. By definition, the refined torsion angle of a double bond is not a crystallographic observation from protein crystals but an interpretation of an observed electron density map; neither do all stereochemical parameters including bond lengths and angles. That is to say, the same experimental map could be interpreted differently. It is a common pitfall to interpret smoothly evolving electron density maps in time-resolved studies as a synchronous conformational change rather than continuous population shifts involving a small number of distinct metastable states. These non-ideal, or even contorted, double bonds most likely resulted from a misinterpretation of *trans*/*cis* mixed electron density maps as a single conformation. Structural refinement software based on least squares fitting would have no better choice but produces an odd torsion angle as a compromise between two distinct states.

Perhaps, this important point can be better explained using spectroscopic data. Similar to Figs. 2(b), 3(a), and 3(b), both PYP and bR also showed smooth and gradual color changes in time series of absorption spectra and during pH titration (Meyer, 1985; Meyer *et al.*, 1987; and Oesterhelt and Hess, 1973). Clearly, such smoothness in color change must not be interpreted as a continuous change in torsion angle of a specific double bond passing 90° during isomerization. It is equally wrong to presume that all C_13_=C_14_ double bonds pedal synchronously throughout an ensemble of molecules. From the commonly accepted view of molecular dynamics, the *trans* and *cis* configurations of a double bond correspond to two energy wells narrowly centered around the torsion angle of 180° and 0°, respectively. The smooth transition displayed in absorption spectra results from the gradually falling population of the initial state and the gradually rising population of the end state. The populations of a few distinct states remain mixed at all times. Conformational heterogeneity is evidenced from the temporal smoothness. Similarly, the smooth evolution of electron density maps (see, for example, Figs. S3 and S8 of Nogly *et al.*, 2018) also resulted from relative populational shifts of heterogeneous states. Forceful refinement of a single conformation against a dataset of mixed states would inevitably lead to an erroneous conclusion.

Why does such a straightforward concept in spectroscopy appear deceptive in crystallography? The cause could have originated from these two seemingly conflicting aspects: On one hand, the crystalline periodicity and symmetry presumes that all unit cells in a single crystal are considered identical. Furthermore, all asymmetric units in a unit cell are related by crystallographic symmetry. As such, an entire crystal contains chemically and conformationally homogeneous molecules. On the other hand, the thermodynamic principle affirms that the reactants, intermediates, and products of a chemical reaction initiated in a crystal would disobey the crystalline periodicity and symmetry, the so-called “time-dependent substitutional disorder” (Ren and Moffat, 1994). These scenarios are analogous to a well-rehearsed military march in formation vs a civilian holiday parade. Moffat asserted that the thermodynamic principle supersedes crystallinity so that “all molecules in the crystal lattice behave independently of one another as if they were in dilute solution” (Moffat, 1989). However, the recent time-resolved studies of bR appear to uphold crystallinity over thermodynamics as the photoisomerization of retinal was depicted as a single dominant intermediate species pedaling around C_13_=C_14_ double bond continuously from *trans* to *cis* and synchronously throughout the crystal. Yet, it is curious why such a cornerstone principle in time-resolved crystallography advocated long ago by Moffat had not been followed nor disputed.

One may argue that refining a single conformation does not exclude the possibility of other coexisting conformations. A theoretically plausible conformation derived from molecular dynamics simulation had often been used to defend the crystallographic refinement result. If a double bond is refined to a torsion angle of ∼90°, it implies that such distorted conformation represents the most dominant population among all possible photoproducts at a given time point, which can only be justified by an energy well near 90° torsion angle upon photon absorption. Yet, no evidence, theoretical or experimental, suggests so. Does each double bond pedal, twist, rotate, and pass the midpoint between *trans* and *cis* at 90° torsion angle? One may imagine that a small fraction of the molecular ensemble could happen to be passing 90° torsion angle during the x-ray exposure. Molecular dynamics simulation could also show such passing of an energy barrier. Nevertheless, a transient conformation at ∼90° torsion angle without an energy well cannot out populate other intermediate species in the well-known energy wells of *trans* and *cis* configurations. In other words, it is not convincing, could even be misleading, to declare a direct observation of a double bond so contorted at near 90° torsion angle solely based on a refinement result in protein crystallography. However, it is in fact not the central point of these studies to claim that an unexpected energy well had been discovered near 90° torsion angle. We will demonstrate below that a straightforward reinterpretation of the observed maps would satisfactorily explain the seemingly twisted double bonds—both *trans* and *cis* are coexisting in the ensemble and only their relative composition is changing.

The same principle also applies to any interpretation of time-resolved electron density maps that capture the rupture or formation of a chemical bond, as in the photolysis of carbonmonoxy myoglobin and hemoglobin. Two atoms are either bonded with a proper bond length or not bonded but engaged in van der Waals contact, or not in contact at all. A refined bond length longer than the proper value but shorter than van der Waals distance strongly suggests a mixture of both bonded and nonbonded species. A claim of direct observation of an unusual bond length in between is very likely erroneous and would require extraordinary evidence beyond structural refinement to prove otherwise.

Do these two contrasting interpretations, continuity in conformation vs continuity in composition, differ only in semantics? Are they equivalent therefore inconsequential to mechanistic understanding? We conducted a joint SVD analysis on a collection of time-resolved XFEL datasets of bR plus a few more synchrotron datasets (Weinert *et al.*, 2019). By extensively applying the rotation theorem [Eq. (6)], we were able to isolate the concurrent structural events during photoisomerization of retinal along with detailed structural information. Contrasting to “the trajectory of retinal isomerization,” this analysis presents an entirely different story regarding how photoisomerization takes place in bR (Ren, 2022).

Retinal incorporated in bR isomerizes from all-*trans* to 13-*cis* upon photon absorption at ∼500 fs specifically at C_13_=C_14_ double bond with a high quantum yield (Govindjee *et al.*, 1990; Tittor and Oesterhelt, 1990). In contrast, photoisomerization of all-*trans* retinal is slow and sluggish in an organic solvent or in the gas phase. Isomerization to 11-*cis* is preferred in solution, but other double bonds are also allowed to isomerize although with poor quantum yields (Freedman and Becker, 1986; Hamm *et al.*, 1996; Koyama *et al.*, 1991; and Logunov *et al.*, 1996). Here the deconvoluted structural events explain how the interactions between the retinal chromophore and its protein environment catalyze the productive pathway of isomerization but shut down the other pathways to unwanted byproducts by tuning the potential energy surfaces of the ground and excited states. At the ground state, the all-*trans* retinal, including the *trans* C_15_=N_ζ_ double bond of the Schiff base, adopts a rather flat geometry [Fig. 10(c)]. Both experimental and theoretical studies suggested that a charge separation is established between the *β*-ionone ring and the Schiff base upon photon absorption (Mathies and Stryer, 1976; Nogly *et al.*, 2018; and Schenkl *et al.*, 2005). Their data allowed us to estimate that the separated charge is equivalent to a fraction of an electron thus a pair of attraction forces of several piconewton are exerted on the *β*-ionone ring and the Schiff base toward each other (Ren, 2022). This photoinduced Coulombic attraction is an intrinsic property of all-*trans* retinal, regardless of whether it is incorporated in protein and in which protein. As the flat plate of the retinal becomes subjected to a longitudinal compressive force at the Frank–Condon point, that is, 0+ time point, the flat retinal has to be compressed and develop some curvature, which was exactly observed in our deconvoluted maps at ∼30 fs. Our intermediate structures refined against the deconvoluted maps show that the retinal molecule is shortened and curves into a wavy S shape (Fig. 2 of Ren, 2022). The S-shaped retinal is the consequence of twisting all single bonds from C_6_ to C_15_ along the polyene chain under compression [Fig. 10(c)]. The largest twist exceeds 60° from the ideal *anti* conformation of 180°, which occurs early in the photocycle and near the *β*-ionone ring. The twists subside gradually and diminish completely at a rather late L state [Fig. 1(c) of Ren, 2022]. Such conformational continuity reflects little variation in the energy landscape throughout the torsional rotation of a single bond in contrast to configurational discreteness of a double bond. All double bonds remain in near-perfect *trans* configuration except that C_13_=C_14_ flips to a near-perfect *cis* configuration before reaching the J state. We did not find any double bond twisted at the midpoint of isomerization necessary for satisfactory map fitting. This study demonstrates that the same crystallographic datasets can be interpreted differently depending on whether mixed structural signals are properly deconvoluted.

Although no double bond was found deviating from the near ideal *trans* and *cis* configurations, we postulated that all double bonds along the polyene chain attempt to sample the possibilities of isomerization, but to no avail, except one [Fig. 10(c)]. The common consequence of these attempts was directly observed as a transient expansion of the retinal binding pocket before a successful isomerization [Figs. 10(a) and 10(b)]. The failed isomerization attempts, and restricted single bond twists are due to the same reason—the retinal is tightly boxed in its binding pocket of the protein. The most constrictive site centers at the middle segment of the polyene chain, allowing larger twists toward the *β*-ionone ring and the only successful isomerization at C_13_=C_14_ double bond [Fig. 3(a) of Ren, 2022]. Without this binding pocket, a free all-*trans* retinal in solution is allowed to isomerize at multiple double bonds, but preferably to 11-*cis* as the C_11_=C_12_ double bond is located at the midpoint of the polyene chain. All isomerization quantum yields in solution are poor because single bond rotation is energetically easier. A *cis* double bond and a *syn* single bond would deliver the same outcome—bringing the *β*-ionone ring and the Schiff base toward each other—except that a *syn* single bond would revert to *anti* spontaneously after charge recombination but a *cis* double bond would not. Recently, multiple isomerizations from all- *trans* retinal captured during the early photoreaction of another microbial rhodopsin are likely incomplete isomerization attempts driven by the same Coulombic attraction force before settling to a complete isomerization to 11- *cis* (Kaziannis *et al.*, 2024). As the protein environment of retinal in bR hinders many pathways to the wasteful byproducts, the productive quantum yield to 13-*cis* is greatly enhanced because it is the only remaining pathway. The consequence of such tuning of potential energy surfaces was also visualized as one of the SVD components ***R***_10_ resulting from the rotation theorem [Eq. (6) and Fig. 10(a)]. This component map can be readily modeled by the retinal binding pocket expansion before isomerization [Fig. 10(b)]. The very early pocket expansion event at hundreds femtosecond strongly suggests that the retinal is sampling numerous options of isomerization along with single bond twisting. Although these attempts manage to push the binding pocket outward quite a bit, the elastic strength of the transmembrane helices imposes strong stereochemical restraints on the retinal chromophore, thereby imposing insurmountable energy barriers to suppress any byproducts, hence catalyzing the intended product. Several mutants at the retinal binding pocket fail to do so, which permits by-product formation to various extents, therefore impairing the pumping activity (Ren, 2022).

This study of photoisomerization in bR demonstrates that heterogeneous structural deconvolution offers mechanistic insights into the catalysis of a chemical reaction that cannot be obtained from electron density maps of mixed states. We must point out that the deconvolution is not limited to the separation of distinct conformational species, it also allows the separation of structural events. Here, retinal isomerization and its binding pocket expansion are two structural events that may or may not concur. As the deconvolution formulated in Eq. (3), each observed electron density map *ρ*(***X***, ***r***) or difference map Δ*ρ*(***X***, ***r***) is expressed as a linear combination of several maps of pure conformational species. When this linear combination is derived from SVD and the subsequent rotation [Eq. (5)], the components involved in the linear combination may or may not represent pure conformations. Instead, they could depict distinct structural events. It is equally informative, if not better, to present the I state of bR as a conformational state of all-*trans* retinal linearly combined with an expanded binding pocket, the precursor of the J state as 13-*cis* retinal combined with a recovered binding pocket, and the J state as 13-*cis* retinal in a contracted binding pocket (Ren, 2022). In other words, concurrent structural events taking place in different regions of a protein, such as isomerization of the retinal and its binding pocket expansion, are more likely to be orthogonal to one another, hence representing more elemental components compared to those depicting pure conformational species throughout a chemical reaction.

### Electronic orbital transitions

Structural signals captured by x-ray crystallography are derived from detected x-rays scattered by the electrons of protein, prosthetic cofactors, and solvent molecules in a crystal. A protein with a molecular mass of *m* in Da contributes at least *m*/2 electrons to scatter x-rays. Each of these electrons resides strictly in its own orbital, that is, a probability distribution with a specific shape depending on its quantum state, although this electronic orbital could be extensively delocalized. In essence, a chemical reaction involves electron transfer from one orbital to another. This quantized orbital transition is in theory observable by crystallography as two distinct orbitals before and after the transition would scatter x-rays differently. However, it has been long deemed infeasible in protein crystallography, despite being highly desirable, because the electrons involved in the orbital transitions of a chemical reaction add up to a tiny fraction of *m*/2 electrons that contribute to the total scattering. It is widely believed that crystallographic observations of electron density distribution of a single or several individual electrons would require ultrahigh spatial resolution such as those demonstrated by charge–density analyses that map out bonding electrons in small molecule crystallography (Coppens, 1998; Koritsanszky and Coppens, 2001). For two different systems, we were able to resolve electron density maps that depict the images of orbital transitions in the respective protein environments. Such visualization of orbital transitions involving a small number of electrons is only made possible by deconvolution of signals and systematic errors from different sources.

It is well established that upon photolysis of carbonmonoxy myoglobin (MbCO), the bound CO ligand dissociates efficiently from an iron –porphyrin–imidazole system. For the heme Fe(II) bonded to the CO ligand, the five 3*d* orbitals are split into two energy levels so that its six 3*d* electrons occupy three orbitals at the lower energy level in pairs and leave the other two orbitals empty, resulting in a low-spin state *S* = 0. As the sixth ligand of CO is lost upon photolysis, the dissociated ligand parks at a nearby docking site, and the heme iron returns to its high-spin state *S* = 2 by occupying all of its 3*d* orbitals (Perutz, 1979). Theoretical calculation explained how the newly occupied 3*d* orbitals break the heme planarity and make the iron drop out of the heme plane (Ugalde *et al.*, 2004). The structural consequences of the spin crossover are highly reproducible and have been repeatedly observed using static and time-resolved crystallography. However, none of the crystallographic maps showed any clear signal of orbital occupations in different spin states. By jointly analyzing a collection of ultrafast MbCO datasets (Barends *et al.*, 2015), one of the rotated SVD components ***U***_4_ (no differentiation between notations **U** and **R**) revealed a difference Fourier map that resembles a superposition of several 3*d* orbitals of the heme Fe(II) (Ren, 2019). This component features a network of positive densities in the shape of a cubic cage around the iron with eight peaks at the corners of the cube, a feature indiscernible in any of the raw difference maps (Fig. 3 of Ren, 2019). The positive cubic densities suggest a net gain of electron density distribution while the five 3*d* orbitals are all occupied when the sixth ligand is ejected by photolysis. This crossover of the heme Fe(II) from low- to high-spin takes place concurrently with the other events of conformational changes, such as departure of the CO ligand, heme doming, and out-of-plane displacement of the iron. Since the electron density redistribution due to spin crossover is relatively minor compared with atomic motions causing relocation of many tens or even hundreds of electrons, small signals of the spin crossover are completely immersed, hence undetected in the raw difference maps without a numerical deconvolution.

**FIG. 11.**
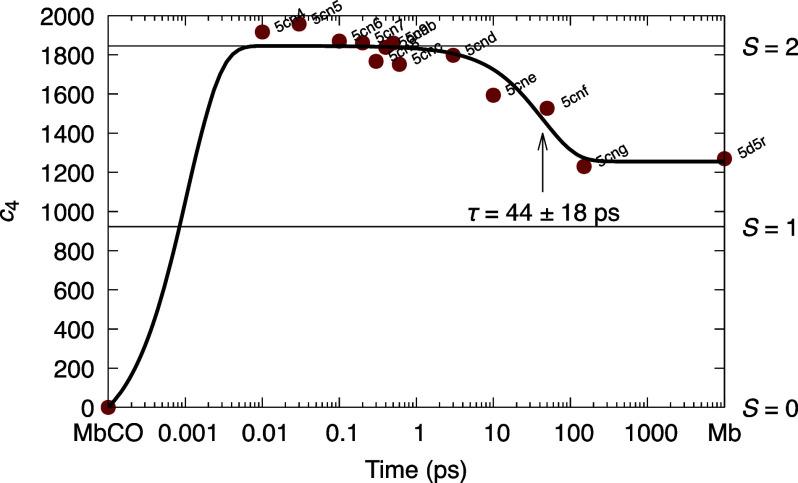
Coefficient *c*_4_ corresponding to the map component revealing spin crossover of heme iron during photolysis of MbCO. The coefficients *c*_4_ rises rapidly from 0 at MbCO, showing spin crossover before 10 fs. A slower decline at 44 ps suggests an equilibrium between the high- and medium-spin states.

In addition, the coefficient *c*_4_ associated with ***U***_4_ decreases monotonically from its maximum at tens femtosecond to ∼2/3 of its peak value as the reaction proceeds for 150 ps, exhibiting an exponential decay with a time constant of 44 ps (Fig. 11). We postulate that this decay arises from ultrafast occupation of the empty 3*d* orbitals before 10 fs followed by a slower transition to a medium-spin state *S* = 1 or an equilibrium between the high- and medium-spin states. This interpretation is supported by theoretical calculations showing that the more energetically favored ground state of an iron –porphyrin –imidazole complex is the triplet spin multiplicity, that is, the medium-spin state *S* = 1, rather than the high-spin state *S* = 2 (Rovira *et al.*, 1997; Ugalde *et al.*, 2004).

**FIG. 12.**
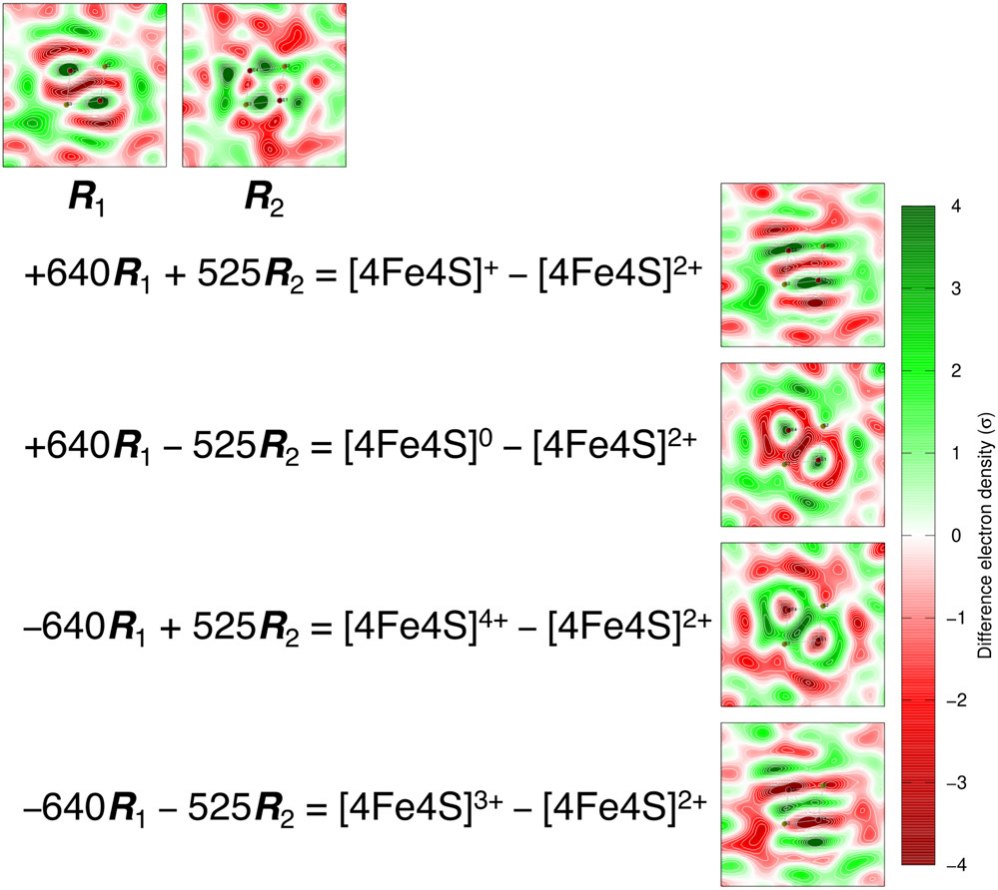
Orbital transitions in [4Fe4S] cluster incorporated in photolyase PhrB. Two most significant component maps ***R***_1_ and ***R***_2_ derived from SVD-based deconvolution are displayed in top panels. Positive and negative densities in one cross section through the iron-sulfur cluster are displayed in green and red respectively. Linear combinations of these component maps result in distinct map features in the same cross section for different valence states from [4Fe4S]^0^ to [4Fe4S]^4+^. The observed datasets show difference maps closely similar to these maps derived from linear combinations, strongly suggesting gain and loss of zero, one, or two individual electrons (Ren *et al.*, 2024). A redox change involving a single electron exhibits layered electronic structure such as [4Fe4S]^+^ and [4Fe4S]^3+^. A two-electron redox change such as [4Fe4S]^0^ and [4Fe4S]^4+^ displays symmetrical electron distribution without layering. These quantized signals originally spanned a subspace involving too many dimensions after the default SVD such that they were not obvious from a random viewpoint. The quantized signals can only be revealed when a proper rotation is applied.

In a recent study, we reported extensive observations of orbital transitions in a four-iron-four-sulfur cluster ([4Fe4S]) incorporated in the DNA repair photolyase PhrB mentioned above (Ren *et al.*, 2024). Our spectroscopic studies showed that the redox changes in the [4Fe4S] cluster are coupled to the interdependent photoreduction reactions of two organic cofactors simultaneously (Ren *et al.*, 2023). According to quantum chemical calculations, two parallel faces of the cubic shape of the iron–sulfur cluster are unique among the six cubic faces due to the coupling of spin momenta of electrons between nearby metal centers. When ferrous and ferric ions coexist in the same cluster, they adopt a mixed valence of Fe^2.5+^ as a result of delocalization of 3*d* electrons (Noodleman and Case, 1992; Voityuk, 2010). Hence, the iron–sulfur cluster was theoretically predicted to have a layering feature in its electron density distribution. Remarkably, such layered electronic structure arising from the redox response of the iron–sulfur cluster has been uncovered by our SVD-based deconvolution of experimental difference maps (Fig. 12). We captured quantized electron density changes with characteristic molecular symmetries as [4Fe4S]^2+^ in the ground state is reduced or oxidized to four other valence states. In addition to those features centered on irons, the captured positive or negative peaks of electron density changes associated with all eight sulfurs, including Sγ atoms of the Cys anchors, suggest electron relaxation of the passive orbitals. We also observed several other important electronic events of orbital transition. Specifically, the valence isomers of [4Fe4S]^2+^ exhibit different orientations of the valence layers; and the iron–sulfur cluster appears to expand in size as it gains sufficient electrons to form a neutral cluster of [4Fe4S]^0^ (Ren *et al.*, 2024).

As demonstrated in both MbCO and PhrB, it is possible to visualize electron density changes due to quantized electronic events if such small changes can be effectively isolated from many other concurrent conformational changes involving atomic displacements. Contrary to the common belief, sharp images at subatomic resolution are not necessary. This is because the probability distribution functions of electrons do not exhibit a mathematical discontinuity that could only be properly depicted by high-frequency terms in the Fourier summation, that is, ultrahigh spatial resolution. Given the resolving power of the SVD-based deconvolution, signals and systematic errors from multiple sources can be isolated, from which weak signals of orbital transition could be extracted. In the absence of significant atomic displacement, electronic movements could stand out as a few top SVD components.

## CONCLUDING REMARKS

On this special occasion that celebrates the work and achievements of Keith Moffat, we felt compelled to revisit a key concept in time-resolved studies—structural heterogeneity. At the dawn of time-resolved crystallography, Keith laid a cornerstone by articulating that the inherent heterogeneity in a chemical reaction can be exploited for studying reaction mechanism without deliberate trapping. With recent developments in serial femtosecond crystallography at x-ray free electron lasers, it becomes ever more relevant and urgent to reiterate this concept in light of the current practice in the field. We were fortunate to join Moffat's early endeavor to capture protein dynamics by crystallography. Thanks to those struggles and failures, we have been striving for experimental and analytical innovations in our independent careers. In this review, we present the formulation, utility, and outcome of SVD-based deconvolution that enables isolation of distinct molecular events from heterogeneous experimental data. As we have demonstrated in diverse systems, the resolution of structural heterogeneity in dynamic data plays a key role in gaining new insights into some of the most fundamental biochemical processes.

## Data Availability

The data that support the findings of this study are available from the corresponding authors upon reasonable request.
